# Development of a Multiple Loci Variable Number of Tandem Repeats Analysis (MLVA) to Unravel the Intra-Pathovar Structure of *Pseudomonas syringae* pv. *actinidiae* Populations Worldwide

**DOI:** 10.1371/journal.pone.0135310

**Published:** 2015-08-11

**Authors:** Serena Ciarroni, Lorenzo Gallipoli, Maria C. Taratufolo, Margi I. Butler, Russell T. M. Poulter, Christine Pourcel, Gilles Vergnaud, Giorgio M. Balestra, Angelo Mazzaglia

**Affiliations:** 1 Department of Science and Technology for Agriculture, Forestry, Nature and Energy (DAFNE), University of Tuscia, Viterbo, Italy; 2 Department of Biochemistry, University of Otago, Dunedin, New Zealand; 3 Institute for Integrative Biology of the Cell, CNRS, Univ. Paris-Sud, Université Paris-Saclay, Orsay, France; 4 ENSTA ParisTech, Université Paris-Saclay, Palaiseau, France; Institut National de la Recherche Agronomique, FRANCE

## Abstract

The bacterial canker of kiwifruit by *Pseudomonas syringae* pv. *actinidiae* is an emblematic example of a catastrophic disease of fruit crops. In 2008 a new, extremely virulent form of the pathogen emerged and rapidly devastated many *Actinidia* spp. orchards all over the world. In order to understand differences in populations within this pathovar and to elucidate their diffusion and movements on world scale, it is necessary to be able to quickly and on a routine basis compare new isolates with previous records. In this report a worldwide collection of 142 strains was analyzed by MLVA, chosen as investigative technique for its efficacy, reproducibility, simplicity and low cost. A panel of 13 Variable Number of Tandem Repeats (VNTR) loci was identified and used to describe the pathogen population. The MLVA clustering is highly congruent with the population structure as previously established by other molecular approaches including whole genome sequencing and correlates with geographic origin, time of isolation and virulence. For convenience, we divided the VNTR loci in two panels. Panel 1 assay, using six loci, recognizes 23 different haplotypes, clustered into ten complexes with highest congruence with previous classifications. Panel 2, with seven VNTR loci, provides discriminatory power. Using the total set of 13 VNTR loci, 58 haplotypes can be distinguished. The recent hypervirulent type shows very limited diversity and includes, beside the strains from Europe, New Zealand and Chile, a few strains from Shaanxi, China. A broad genetic variability is observed in China, but different types are also retrievable in Japan and Korea. The low virulent strains cluster together and are very different from the other MLVA genotypes. Data were used to generate a public database in MLVAbank. MLVA represents a very promising first-line assay for large-scale routine genotyping, prior to whole genome sequencing of only the most relevant samples.

## Introduction

*Pseudomonas syringae* “*sensu latu”* includes nine recognized plant pathogenic species, *P*. *amygdali*, *P*. *avellanae*, *P*. *cannabina*, *P*. *caricapapayae*, *P*. *ficuserectae*, *P*. *meliae*, *P*. *savastanoi*, *P*. *syringae* and *P*. *tremae*, (ISPP Taxonomy of Plant Pathogenic Bacteria Committee, http://www.isppweb.org/names) [1].

The *P*. *syringae* species itself represents a wide complex of different taxa, several of which are pathogens, named pathovars in relation to their host preference. More than 60 pathovars are recognized [2], whilst many other strains genetically referable to *P*. *syringae* are environmental bacteria.

The canker of kiwifruit is caused by one of these pathovars, the Gram negative bacterium *P*. *syringae* pv. *actinidiae* (Psa). It engenders a series of characteristic symptoms, such as brown leaf spots with chlorotic haloes, whitish to reddish exudates on trunks and twigs, sudden death of young vines, collapse of fruits and brown discoloration of buds [3,4]. This pathogen was first isolated, recognized as causal agent of the bacterial canker of kiwifruit on *Actinidia deliciosa* and described as a new pathovar in Japan in 1984 [5]. Almost contemporaneously, the disease was described in Hunan province, China, in 1984 [6]. Psa has then spread among the main kiwifruit cultivation areas of China such as Anhui, Shaanxi and Sichuan provinces [7–13]. In Korea the disease was reported since the 1990’s [14] up to present time [15–17], whilst in Italy there was an occasional report in 1992 [18]. In 2008 a new, very aggressive type has emerged. It caused serious damage and showed very rapid diffusion mainly on *Actinidia chinensis* [19], but also on *A*. *deliciosa* [3]. In 2010 the pathogen reached orchards of both *A*. *deliciosa* and *A*. *chinensis* in France [20], Portugal [21] and even New Zealand [22]. In 2011 the pathogen was recovered in Switzerland, Spain, Turkey, Australia and Chile [23–27], and the disease is still spreading, as demonstrated by reports from Germany [28], Slovenia [29] and Greece [30].

Since the beginning of the epidemic by the hypervirulent type of Psa in Europe and New Zealand, research efforts have been multiplied and different types of DNA-based analyses have been investigated to genetically characterize the populations of Psa [31]. The first attempts used rep-PCR analysis [32,33] to detect differences among European, Korean and Japanese strains. Subsequently Multiple Loci Sequence Typing (MLST) was applied. In 2012 Chapman *et al*. [34] characterized 40 Psa strains by sequencing seven housekeeping genes (acn, rpoD, gapA, cts, pgi, pfk, gyrB), and eleven putative effector genes and phytotoxins genes. The authors were able to allocate the strains into four biovars: Psa1 defined by five strains isolated in 1984–1988 from Japan and the Italian strain obtained in 1992; Psa2 defined by three Korean strains isolated in 1997–98; Psa3 defined by strains from New Zealand, China, Chile and Italy isolated after 2008; Psa4 defined by a particular group of strains showing a paltry virulence on kiwifruit and isolated only in New Zealand and Australia. The Psa4 low virulence strains are responsible only for foliar spots and do not cause cankers or other symptoms.

Since 2011 several research groups have carried out whole genome sequence (WGS) analysis of Psa [4,35–37]. In 2012 Mazzaglia and colleagues [35] reported the draft genome sequences of nine strains of Psa and one of the pathovar *theae*, the closest known relative to Psa. The three Chinese strains, isolated from Shaanxi province in 2010, and European strains were almost identical, separated from each other by only six single nucleotide polymorphisms (SNPs), but with some differences in a particular genomic island (PPHGI-like). The genomic analysis confirmed that these strains were clearly distinct from Japanese and Korean strains. A derived PCR approach indicated also that the virulent strains from New Zealand belong to the same Chinese/European group. A multiplex PCR assay, using primers specific for European, Chinese and Japanese/Korean strains, allowed both the recognition of virulent Psa from other similar pseudomonads present on kiwifruit, and the assignment of the analyzed strains to the correct haplotype in a simple and accurate way [38].

In 2013, Butler and colleagues [36] investigated the genomes of 23 Psa strains, finding sets of SNPs that were characteristic of strains from Italy, New Zealand or Chile. Together with the analysis of genomic islands (Integrative Conjugative Elements—ICE), the data sustained the hypothesis of an independent Chinese origin for the European, New Zealand and Chilean outbreaks. The authors proposed to exclude the low virulent (“LV”) strains, corresponding to the biovar Psa4, from the pathovar *actinidiae*. In 2015, Cunty et al [39], reported the presence of Psa4 in France. This was the first occurrence out of Australasia. The authors proposed to define a new pathovar, *Pseudomonas syringae* pv. *actinidifoliorum*. To simplify the comprehension of this paper, we will still refer to this group as *Pseudomonas syringae* pv. *actinidiae* biovar 4 (or Psa4).

The 2013 study by McCann and colleagues [37] included 34 draft WGS Psa genomes and two closed genomes. It confirmed the existence of the above cited four Psa lineages, discussed the possibility of recombination between them and suggested that they share a recent common ancestor.

Molecular information about contemporary Japanese and Korean Psa populations has been acquired only recently. Sawada and colleagues [40] reported the presence of a new MLST group isolated in Japan since 2010, named Psa5. Similarly, Koh et al [17] compared two strains isolated in 1999 in Korea with two isolated in 2011 and concluded that currently two populations exists in Korea.

Only limited data are available from China [13], again poorly or not at all comparable with other data. Therefore, further researches and appropriate methods are needed to clarify the diversity in the Psa populations from within the enormous area of *Actinidia* cultivation in China and from where the wild species of *Actinidia* originate.

Although WGS analysis is increasingly presented as the method of choice for such investigations, at least for more academic investigations, providing an enormous source of information and very robust phylogenies [41], it is currently too expensive to be used as a first line assay. It is poorly or not at all applicable in quick monitoring or in analysis of a high number of strains.

Alternatively, polymorphic tandem repeats can be used to constitute solid and powerful first line assays in highlighting reliable differences between individuals of the same taxon and identifying the most relevant strains on which to apply WGS [42–48]. Amplicons obtained by standard PCR using primers designed on the flanking regions of the tandem repeats, are analyzed by regular agarose gel electrophoresis, high resolution electrophoresis techniques (i.e. capillary electrophoresis) or sometimes by high resolution melting curve analysis (HRMA) [49] depending upon the required size estimate precision and available equipment. Tandem repeats with large repeat units can usually be typed with correct results using any of these technologies, whereas tandem repeats with short repeat units and/or large alleles need to be analyzed using capillary electrophoresis. The use of different fluorescent labels allows to multiplex the PCRs.

The use of a collection of VNTRs for typing purposes is called MLVA for Multiple Locus VNTR Analysis. MLVA provides data in a numeric form which can be easily stored in databases accessible via internet such as http://mlva.u-psud.fr [50,51]. Some databases, such as for the *Brucella* MLVA database, contain data from thousands of strains, independently produced by many laboratories.

In Table 1 are summarized some recent study cases of genotyping of phytopathogenic bacteria, including *P*. *syringae*, for which MLVA has shown promising results [52–61].

**Table 1:** Recent epidemiological studies on bacterial pathogens of plants, carried out by MLVA.

Authors	Year	N° of VNTR loci	Pathogen Species	Host plant species
Della Coletta-Filho *et al*. [52]	2001	8	*Xylella fastidiosa*	*Citrus sinensis;Coffea arabica*
Ngoc *et al*. [53]	2009	14	*Xanthomonas citri* pv. *citri*	*Citrus* spp.
Bergsma-Vlami *et al*. [54]	2012	6	*Xanthomonas arboricola* pv. *pruni*	*Prunus laurocerasus*
Gironde and Manceau [55]	2012	8	*Pseudomonas syringae pv*. *maculicola;Pseudomonas syringae* pv. *tomato*	*Brassicaceae* fam.; *Lycopersicon esculentum*
Zhao *et al*. [56]	2012	25	*Xanthomonas oryzae* pv. *oryzicola*	*Oryza sativa*
N’Guessan *et al*. [57]	2013	26	*Ralstonia solanacearum*	*Solanaceae* fam.
Zaluga *et al*. [58]	2013	8	*Clavibacter michiganensis* subsp. *michiganensis*	*Lycopersicon esculentum*
Pruvost *et al*. [59]	2014	31	*Xanthomonas citri* pv. *citri*	*Citrus* spp.
Vernière *et al*. [60]	2014	14	*Xanthomonas citri* pv. *citri*	*Citrus* spp.
Bühlmann *et al*. [61]	2014	6	*Erwinia amylovora*	*Pomaceae* fam.

We here take advantage of published Psa genome sequence data to explore VNTRs and to test them on an extended collection of Psa strains from the areas of the world where the pathogen is already established. By this approach, we investigated differences between Psa strains of different geographical origins, isolation dates and virulence, in order to identify the sources of infection, to help detect any further diffusion and trace the origin of Psa and routes of transmission.

## Materials and Methods

### Bacterial strains and growth conditions

One hundred forty-two Psa strains representative of all the main areas of kiwifruit cultivation where the pathogen has been reported (14 from six different European Countries, one from Turkey, six from New Zealand, three from Australia, 29 from Chile, 22 from Korea, ten from Japan and 57 from China) and isolated during a period of about 30 years, were selected from the bacterial collection of the D.A.F.N.E., University of Tuscia in Viterbo (S1 Table).

Strains were grown on King's medium B (KB) plates at 28°C for 48 hrs before DNA extraction.

### Genomic DNA extraction

The Bacterial Genomic DNA Isolation Kit (Norgen Biotek Corp., ON, Canada) was used to obtain DNA from freshly grown cultures of each of the 142 strains. Each DNA sample was quantified using Qubit Fluorometer (Invitrogen, Life Technologies Italia, Monza, Italy), adjusted to a concentration of 40 ng per μl with TE (10mM Tris-HCl, 1mM EDTA) buffer at pH 8.0, and stored at -20°C until use.

### Tandem repeats identification and design of VNTR primers

A preliminary analysis of the WGS data from three geographically separate Psa strains available in the NCBI database (Italian strain CFBP 7286, Chinese strain CH2010-6 and Japanese strain M302091) was carried out to identify suitable tandem repeats (TRs). The sequence data were imported into The Microorganisms Tandem Repeats Database (http://tandemrepeat.u-psud.fr/) [50] and screened using the following parameters: tandem repeat array from 50 to 1000 bp, repeat unit length from 5 to 300 bp, and >80% similarity within the copies of the tandem repeat array.

Potentially useful VNTRs were selected as those with predicted size polymorphism among the three genomes. Right and left flanking regions of about 100 bp each were used to confirm by BLAST searches that these regions were present in all the WGS sequences available for Psa. Primer pairs were designed in the flanking regions of each selected tandem repeat with Primer3plus (http://www.bioinformatics.nl/cgi-qbin/primer3plus/primer3plus.cgi), aiming to have an average length of 20 bp, a Tm of 59–60°C in order to ensure a high specificity of amplification and to be suitable for use in a prospective multiplex assay.

The Psa genomes were screened with the primer sequences by *in silico* PCR using Primer-BLAST (http://www.ncbi.nlm.nih.gov/tools/primer-blast/) to confirm the amplification of a single product. Primers were synthesized by Macrogen (Macrogen Europe, Amsterdam, the Netherlands).

In addition, we evaluated the panel of eight TRs used by Gironde & Manceau [55] to genotype *Pseudomonas syringae* pv *tomato* and *maculicola*. We renamed the VNTRs from Gironde and Manceau according to their position (rounded to kb) in the chromosome of strain ICMP 18884 of *Pseudomonas syringae* pv. *actinidiae*, adding GM as prefix (i.e. GM-254).

The draft genome sequences of ten of the 142 strains investigated here (marked with * in S1 Table) were previously reported and are publicly available in the WGS database of NCBI. Four of them were sequenced twice (Table 2).

**Table 2:** Strains included in this study whose WGS assemblies are present in NCBI database, their aliases in papers or in international collections, and related accession numbers.

Strain Id	Aliases	Accession numbers	references
PSA_IT92	NCPPB 3871	AFTF	[4]
PSA_KW11	NCPPB 3739, ICMP 9617	AFTH, AOKP	[4,37]
PSA_7286	CFBP 7286	AGNO	[35]
PSA_CH2010-6	M7	AGUH, ANJJ	[35,36]
PSA_PA459	CFBP 5097	AGNQ	[35]
PSA_KW41	ICMP 9855	AGNP, AOKB	[35,37]
PSA_KW1	ICMP 9853	ANJB	[36]
PSA_18804	ICMP 18804	ANJE, AOJU	[36,37]
PSA_19439	ICMP 19439	ANJM	[36]
PSA_M228	-	ANJI	[36]

NCPPB: National Collection of Plant Pathogenic Bacteria (UK).

ICMP: International Collection of Microorganisms from Plants (NZ).

CFBP: Collection Française de Bactéries associées aux Plantes.

Results of MLVA analysis were compared with in silico data from corresponding WGS sequences.

### PCR amplification and screening of VNTR loci

Each PCR reaction was composed of 12.5 μl of GoTaq Colorless Master Mix 2X (Promega Corporation, Madison, WI, USA) containing GoTaq DNA Polymerase, 400 μM of each dNTP, 3mM MgCl_2_, 1 μl of template DNA (40 ng), 1 μl of forward and 1 μl of reverse primer corresponding to 10 μM concentration each, 9.5 μl of nuclease-free water to reach the final volume of 25 μl.

The PCR amplifications were performed on a C1000 thermal cycler (Biorad Laboratories Inc., Ca., USA) with the following conditions: initial denaturation for 5 min at 94°C, followed by 30 cycles of denaturation for 30 s at 94°C, annealing for 30 s at 58°C, extension for 45 s at 72°C plus a final elongation step for 7 min at 72°C.

VNTR loci that gave an identical number of repeats in the ten strains selected as “diversity panel” (marked with † in S1 Table) were discarded. Each of the TRs that showed discriminatory power in this preliminary screening test was then applied to the additional pool of 132 strains of Psa with the same PCR amplification protocol. The whole trial was repeated twice to test the reproducibility of the assay. Between the two trials single Psa colonies were streaked ten times successively to test the stability of the selected VNTRs.

### VNTR analysis by capillary electrophoresis

The QIAxcel capillary electrophoresis system (QIAGEN, Milan, Italy) was used to estimate the amplicons size. For all but one VNTR locus, we applied method OM700, recommended for amplicons up to 500 bp, that uses the High Resolution cartridge and the following run parameters: 10 s of sample injection time; 5 kV of sample injection voltage; 3 kV of separation voltage for 700 s of separation time. Only for the VNTR locus Psa-09, due to its dimension, we applied method OM500, recommended for amplicons up to 1 kb, that is characterized by higher separation voltage (5 kV) and shorter separation time (500 s). Results were analyzed and interpreted by means of the Screengel software (QIAGEN), which gives estimates of both size and concentration of amplicons.

### VNTR analysis and statistics

The repeat copy number convention assignment in case of loci with a truncated repeat was rounded up to the next integer so that strains potentially having a single truncated element will be scored as 1 rather than 0 which might be confused with amplification failure [45].

To confirm the accuracy of repeat copy number assignments, the whole set of 13 VNTR amplicons from three representative strains (PSA_7286, PSA_KW11, PSA_CH2010-6) were sequenced by Sanger sequencing. Furthermore, amplicons for which the number of repeats appeared different from the closest isolates, were confirmed by Sanger sequencing.

The discriminatory power of each VNTR locus was assessed by calculating the Simpson’s Diversity Index [62] using the online tool V-DICE (http://www.hpa-bioinformatics.org.uk/cgi-bin/DICI/DICI.pl).

The Bayesian clustering software STRUCTURE version 2.3.4 [63–66] was used to infer the overall population structure. Following the recommendations reported in Evanno et al. [67], we carried out 20 independent runs for each of the putative number of populations (K), from 1 to 13, using 750000 burn-in replicates and 500000 MCMC (Markov Chain Monte Carlo) simulations per each run. STRUCTURE was run with the following options: admixture model, that assume correlation between alleles frequencies of data, without prior population information. The posterior probability of the data for a given K was calculated and labeled as L(K).

The MLVA data were analyzed as a “character” dataset to draw minimum spanning trees (MSTs) using BioNumerics version 7.5 (Applied Maths NV). Dendrograms were drawn using DARwin 6 software [68,69] and a similarity matrix calculated by Simple Matching Dissimilarity Index (Sokal-Michener Index) for categorical data, with 1000 bootstrap iterations. Strains were clustered hierarchically with the Unweighted Neighbor-Joining (UNJ) algorithm [70].

## Results

### VNTR loci

Eleven potentially effective VNTR loci, Psa-01 to Psa-11 were identified from the *in silico* analysis.

Together with the eight loci previously reported by Gironde & Manceau [55], they were tested for their discriminatory power using the “diversity panel” of ten Psa strains, chosen for being isolated from different infection sites in the world and at different times.

Six loci, two from our selection and four from the panel used by Gironde & Manceau, were monomorphic in the “diversity panel” and were not further explored. The 13 other VNTR loci were tested on the whole Psa panel of 142 strains. The primers for PCR amplification of these VNTR loci, the position of the corresponding amplicons on the circular chromosome of ICMP 18884 (accession number NZ_CM002751.1) and, when present, the putative function of overlapping genes are indicated in Table 3. The tandem repeats in 10 out of 13 cases fall within intergenic sequences (reported as unknown) of the reference genome used. In Psa-09 and Psa-10 the repeats are included partially in hypothetical proteins. Only in the MLVA locus GM-1834 the tandem repeats are totally included in a gene coding for a specific protein, and, as expected for intragenic loci, the repeat unit has a size of 6 bp, multiple of 3 bp.

**Table 3:** Arrangement of each VNTR locus on genome of *Pseudomonas syringae* pv. *actinidiae* strain ICMP 18884.

MLVA	*Pseudomonas syringae* pv. *actinidiae* ICMP 18884 chromosome (annotated WGS sequence—accession NZ_CM002751 AOKO01000000)	
VNTR name and related primers (5’→3’)	ORFs	Putative function of the ORFs
**Psa-01**F: CAAGCAGGAGATGGAAGAGCR: CATGCGGGCAATCTGATAGT	3007109–3007161	end of “LysR-type transcriptional regulator CysB” gene
	3007162–3007280	unknown
	3007281–3007310	beginning of “phosphoadenosine phosphosulfate reductase” gene
**Psa-03**F: TTATCGGCGGGATGTGTATTR: ACTGCGTCTGGTCGATAACC	874124–874146	end of “iron-uptake factor” gene
	874147–874274	unknown
	874275–874338	beginning of “peptide ABC transporter ATP-binding protein” gene
**Psa-04**F: ACGAGTCCGCTCCTACAAAAR: TACAACCAAGGTGGCCTGTT	2105844–2106066	unknown
**Psa-05**F: GTAGGCCGCGCTTTCAATR: TGCACTTCTTTTTCGCCTCT	5506545–5506733	unknown
**Psa-06**F: GCCTACCTTTTACGCCATGAR: TTAACGCAAGCAATCCTAACC	742145–742298	unknown
**Psa-07**F: TGTGCAATAAATGCGGGTTAR: CCGCCTCCAGTCAGGTTAAT	742264–742335	unknown
**Psa-08**F: GTCATTGGCGAACTGATCCTR: CTTTTCATGCTGAAAGTCATGC	1302522–1302545	end of “hypothetical protein” gene
	1302546–1302692	unknown
**Psa-09**F: CGCTGTCTGGCTTTGAAAATR: TAGGACGGCCGAAGGTTTAT	982509–982524	end of “transposase” gene
	982525–982566	unknown
	982567–982797	“hypothetical protein”
	982798–982840	unknown
	982841–983062	“hypothetical protein”
	983963–983129	unknown
**Psa-10**F: AAGCCTGAGTAAGCGGTTCAR: GCCCCAGTCCCAGTTGTAAT	2399774–2399797	end of “amino acid ABC transporter permease” gene
	2399798–2399831	unknown
	2399832–2399931	“hypothetical protein”
	2399932–2400012	unknown
	2400013–2400019	beginning of “ABC transporter” gene
**GM-254**F: CGTGTCACTGAAGTCACCATR: TATTTACCCGGTGTTGAGGC	254532–254617	end of “transposase” gene
	254618–254898	unknown
**GM-1553**F: CTGGCACGAGACGAGTCCR: GCTGAGCTTGAAGGAGACG	1553396–1553473	end of “phosphoriboslglycinamide formyltransferase 2” gene
	1553474–1553562	unknown
	1553564–1553601	beginning of “citrate-proton symport" gene
**GM-1834**F: CGAGTTCTATTTGCGTCAGGR: TGTCCAGCGTAATCTTGCTC	1834304–1834581	Enclosed in "tellurium resistance protein TerA" gene
**GM-4076**F: TGGGTGGAATACAGCCGCCAR: CTTGTTCGGGAGCGGCAAGCT	4076304–4076431	unknown
	4076432–4076494	beginning of “glutamine synthetase" gene

The VNTR loci were named as recommended by [44,45,46] to include the full allele coding convention: *name of the locus_length of repeat unit in bp_in silico length of the whole amplicon in bpin the reference genome_assigned number of repeats (convention including partial final repeat) in U*, according to a specific genome. In our assay, for instance, *Psa-01_7bp_202bp_9U* means that locus Psa-01 has a 7 bp long repeat unit, the whole amplicon is 202 bp in ICMP 18884 accession number NZ_CM002751.1 sequence data when using the primers indicated in Table 3 and by convention this allele is called 9U (Table 4).

**Table 4:** Main features of each VNTR locus used to create the MLVA panel to infer the analysis of the 142 Psa strains.

VNTR locus name including coding convention	TR length	Length of final partial TR	Simpson’s Diversity Index	N° of different alleles	Amplicons range (bp)	Observed alleles(including partial final repeat)
**Psa-01_7bp_202bp_9U** ^a^	7	3	0.746	6	153–202	3–7;9
**Psa-03_7bp_215bp_10U** ^b^	7	4	0.732	10	164–236	3–4;6–13
**Psa-04_33bp_223bp_3U** ^c^ ^f^	33	-	0.378	4	157–223	Ø;1–3
**Psa-05_7bp_189bp_6U**	7	4	0.713	6	161–203	2;4–8
**Psa-06_8bp_155bp_4U** ^e^	8	-	0.443	6	139–171	Ø;2–6
**Psa-07_8bp_72bp_2U** ^e^	8	7	0.417	4	72–88	Ø;2–4
**Psa-08_9bp_171bp_3U**	9	-	0.739	8	162–243	2–4;6–10
**Psa-09_105bp_621bp_6U** ^e^	99–112^d^	32	0.596	4	305–723	Ø;3;6–7
**Psa-10_7bp_246bp_14U**	7	6	0.843	12	162–260	2;6–14;16–17
**GM-254_8bp_367bp_5U** ^e^	8	3	0.429	8	367–423	Ø;4–8;11–12
**GM-1553_7bp_206bp_6U**	7	6	0.741	5	185–220	3–6;8
**GM-1834_6bp_278bp_18U**	6	4	0.855	13	206–278	6–18
**GM-4076_7bp_191bp_2U**	7	6	0.364	3	191–205	2–4

^a^ the low virulence strains have a seven nucleotides gap in right flanking region when using the indicated primers.

^b^ the low virulence strains have a two nucleotides gap in left flanking region when using the indicated primers, resulting in a 164 bp allele.

^c^ the low virulence strains have one additional nucleotide in right flanking region when using the indicated primers.

^d^ the length of TRs can change in the described range.

^e^ these loci are not amplified on DNA from low virulent strains.

^f^ this locus is not amplified on DNA from Japanese strains from 1984 to 1988 and from Italian strain isolated in 1992.

^Ø^ indicates amplification failure.

Eight loci yielded a unique amplicon in all strains. A failure in amplification was observed at locus Psa-04 on six Japanese strains isolated from 1982 to 1988, as well as the Italian strain isolated in 1992. Loci Psa-06, Psa-07, Psa-09 and GM-254 failed to amplify the Psa4 “Low Virulence” strains investigated from NZ and Australia. Locus Psa-09 also failed to amplify 15 Korean strains (see footnotes, Table 4). One hundred and fifty-nine alleles were sequenced as controls to check the exact amplicon size. A string of 13 integer numbers representing the repeat copy number of each VNTR, in which the lack of amplification was coded as “-1”, was defined for each strain (S1 Table).

Sanger sequencing allowed to detect several single nucleotide polymorphisms in the flanking regions of Psa4 “Low Virulence” strains with no impact on allele size and consequently on MLVA analysis (data not shown). In contrast very few differences were found in amplicons from other Psa strains. The left flanking region of VNTR GM-254 is 133 bp long in European, Chinese, Chilean and New Zealand virulent strains, whilst it is 125 bp long in Japanese and Korean strains. This flanking 8 bp microdeletion could be conveniently coded as a VNTR variation since the VNTR has a 8 bp repeat unit.

MLVA data confirmed by Sanger sequencing were compared with the number of repeats as obtainable from WGS for those ten strains of our selection for which genome sequence data are available in Genbank (Table 5). For four of them two independent WGS sequences are available. Several differences were detected and noteworthy the highest number of these discrepancies was often consistent with the highest Simpson’s diversity indices of the loci. Locus GM-1834 with the highest Simpson’s Diversity Index (0.855) also showed the highest number of discrepancies in MLVA-WGS data comparisons (ten among 13 genomes).

**Table 5:** Comparison of the number of TR units for each VNTR locus as obtained from MLVA capillary analysis, confirmed by Sanger sequencing in all cases, and in silico analysis of the corresponding WGS sequences deposited in Genbank.

Strain ID	*Genbankaccession*	Psa-01	Psa-03	Psa-04	Psa-05	Psa-06	Psa-07	Psa-08	Psa-09	Psa-10	GM-254	GM-1553	GM-1834	GM-4076
**CFBP 7286**	*GCA_000245415*.*1*	9(7)	10(7)	3(2)	6	4	2	3	6(n.a.)	6	5	6	17(7)	2
**CH2010-6** ^**a**^	*GCA_000245475*.*1*	9	11(10)	3	5	4	2	3	6	14(10)	5(3)	6	18(11)	2
**M7** ^**a**^	*GCA_000344495*.*1*	9	11(n.a.)	3	5	4	2	3	6	14(n.a.)	5(3)	6	18(n.a.)	2
**PA459**	*GCA_000245455*.*1*	4	4	-	5	3	2	4	7	16(10)	12(n.a.)	4	14(11)	4
**KW41** ^**b**^	*GCA_000245435*.*1*	4	3	-	4	3	3	10(7)	7(n.a.)	14(8)	12	4	14(10)	3
**ICMP 9855** ^**b**^	*GCA_000416665*.*1*	4	3	-	4	3	3	10(36)	7(n.a.)	14(52)	12(10)	4	14(9)	3
**NCPPB 3739** ^**c**^	*GCA_000233835*.*2*	4	3	-	4	3	3	10(n.a.)	7(n.a.)	14(13)	12	4	15(11)	3
**ICMP 9617** ^**c**^	*GCA_000658965*.*1*	4	3	-	4	3	3	10	7(n.a.)	14	12	4	15	3
**NCPPB 3871**	*GCA_000233795*.*2*	3(4)	3	-	4	3	3	10	7(n.a.)	14	12	4	15(14)	3
**ICMP 9853**	*GCA_000344335*.*1*	4	3	-	4	3	4(n.a.)	10	7(n.a.)	14	12	4	15	3
**ICMP 18804** ^**d**^	*GCA_000344395*.*1*	3	3	1	2	-	-	2	-	2	-	3	7	2
**ICMP 18804** ^**d**^	*GCA_000416905*.*1*	3	3	1(n.a.)	2	-	-	2	-	2	-	3(n.a.)	7	2
**ICMP 19439**	*GCA_000344555*.*1*	9	9	3	6	4	2	3	6	11	5(3)	6	17(n.a.)	2
**M228**	*GCA_000344475*.*2*	5	7	3	6	5	2	9	7(n.a.)	9(8)	4	4	16	2

Single numbers point out an exact correspondence between the two values, whilst incongruities are reported with a second number in brackets, e.g. 17(7) means that both MLVA capillary electrophoresis and Sanger sequencing indicate a value of 17 whereas the WGS assembly indicates a value of 7 repeats. In most cases, the MLVA/Sanger result is longer than the WGS result, as expected from the assembly of perfect tandem repeats when the WGS sequencing reads are too short. The lettering n.a. means the lack of the complete corresponding sequence on a single WGS scaffold or contig. In four among ten strains, two independent WGS sequence assemblies are available.

^a^ these are the same strain that was independently sequenced as CH2010-6 in [35] and as M7 in [36].

^b^ these are the same strain that was independently sequenced as KW41 in [35] and as ICMP 9855 in [37].

^c^ these are the same strain that was independently sequenced as NCPPB 3739 in [4] and as ICMP 9617 in [37].

^d^ these are the same strain that was independently sequenced as ICMP 18804 in both [36] and [37].

### Arrangement of VNTRs in sets for analysis and main haplotypes grouping

**Fig 1:**
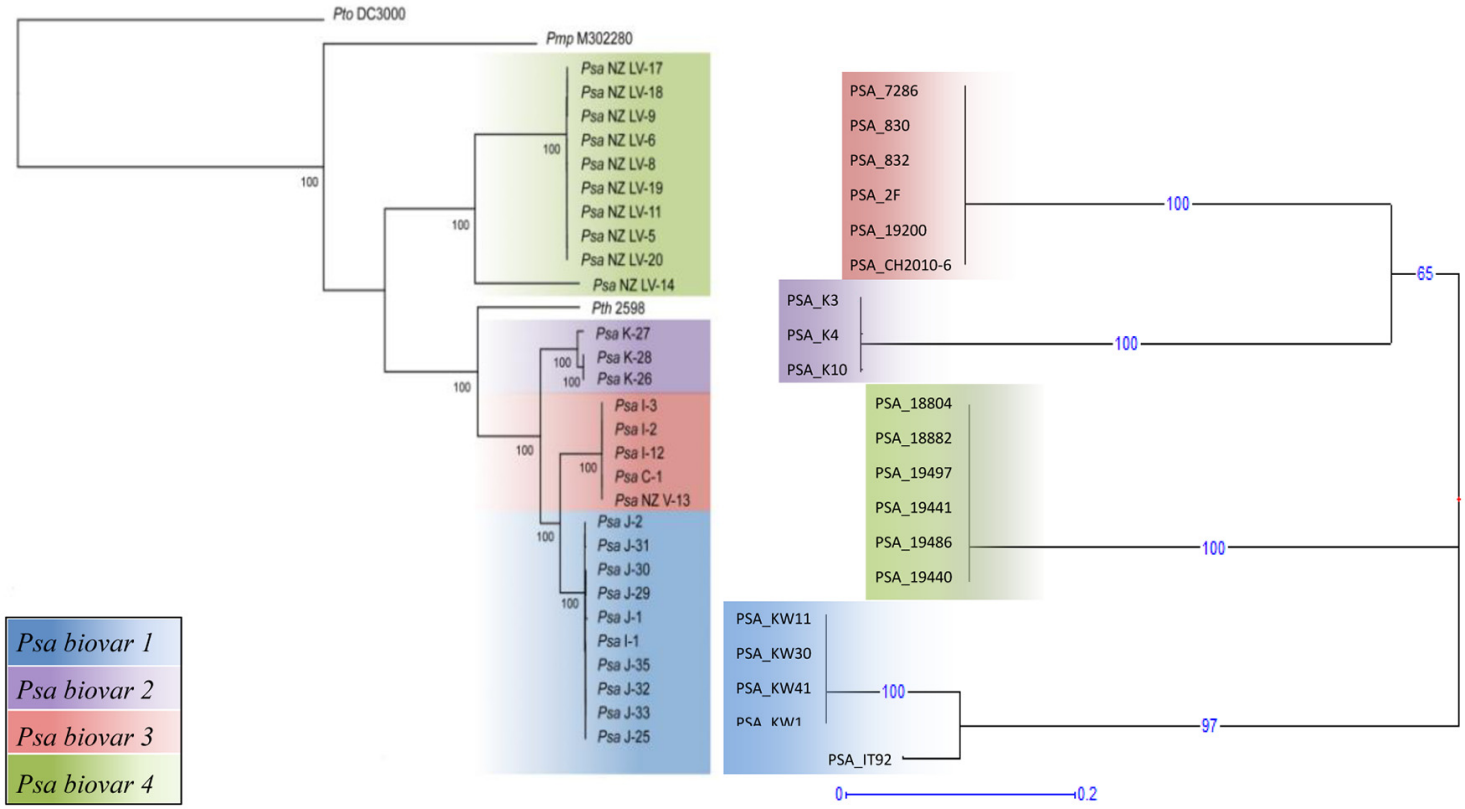
Comparison of dendrograms obtained by SNPs and MLVA analysis. The dendrogram on the left was obtained by Maximum Likelihood analysis on 15,329 SNPs from WGS sequences of 28 Psa strains [37], whilst the radial dendrogram on the right was obtained by Unweighted Neighbor Joining tree from six selected VNTR loci (Panel 1) on 20 strains used in this study selected for having geographical and time origins identical or corresponding to the first ones. Numbers on branches represents the percentage of bootstraps on 500 replications.

We then used the MLVA dataset derived from the 142 strains to identify a panel of loci able to correctly assign strains to the four Psa biovars in agreement with previous studies and current knowledge. We propose a set of six loci, Panel 1, composed of Psa-01, Psa-04, Psa-07, GM-254, GM-1553 and GM-4076). Fig 1 shows a comparison between the dendrogram obtained by McCann et al [37] based upon more than 15000 SNPs from draft genomes of twenty-eight Psa strains belonging to the four known biovars and the dendrogram obtained using Panel 1 on twenty strains selected in order to have geographic and time origins identical or equivalent to those of the strains considered in the dendrogram of the paper of McCann et al [37].

**Fig 2:**
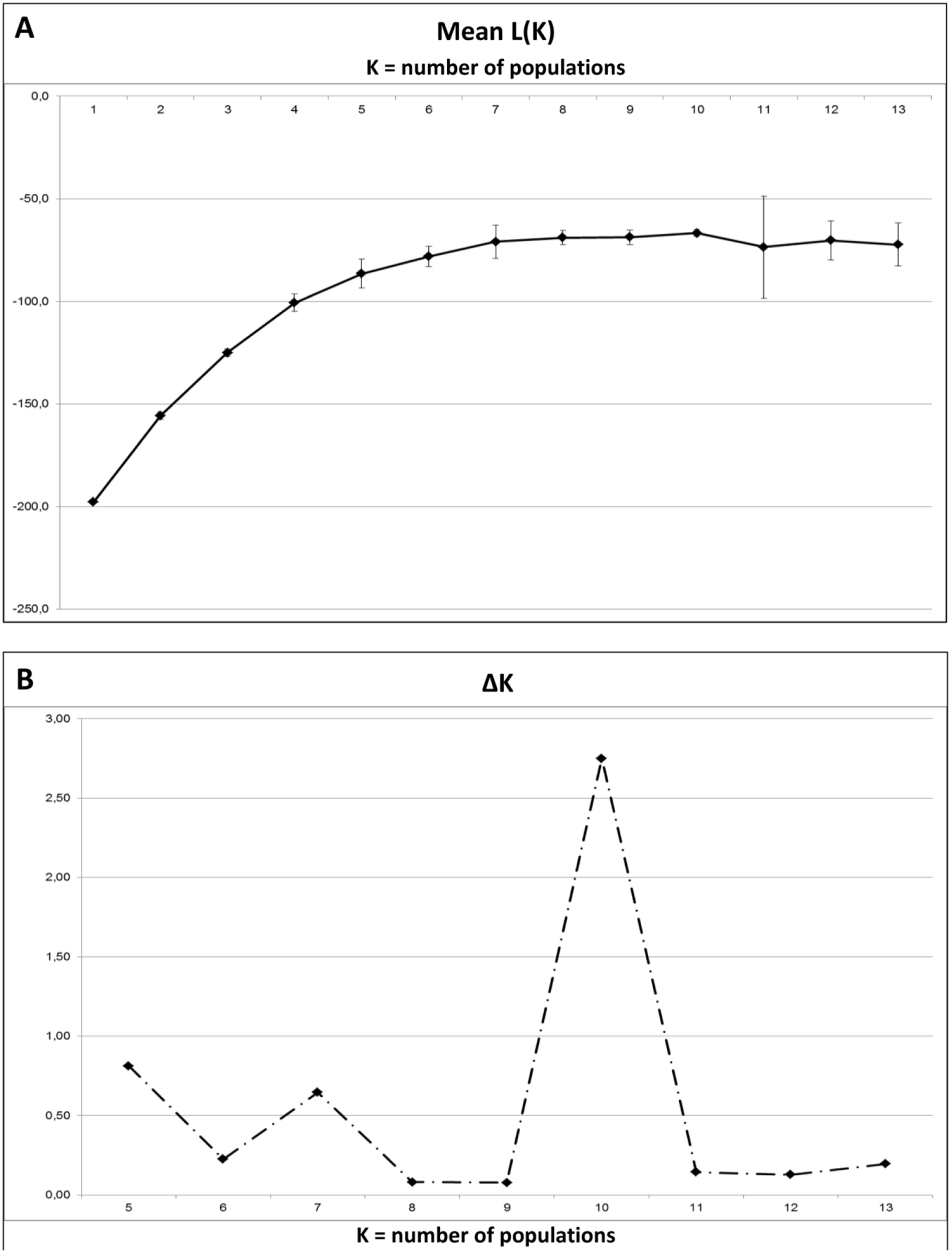
Graphical representation of the main steps to infer the number of populations K. Results obtained by STRUCTURE 2.3.4. (A) Mean L(K) ± SD over 20 runs for each K value. A hierarchical island model was applied on all the 142 strains and the six VNTR loci constituting Panel 1. (B) ΔK = m|L”(K)|/s[L(K)] gives the modal value of the distribution that represents the suggested number of subpopulations among the examined strains, here ten groups.

Panel 1 data from the whole collection of strains was therefore used to evaluate the presence of additional populations using the same criterion. In the STRUCTURE analysis (Fig 2)the estimate of ln[Pr(X K)] reaches a plateau as K comes near to this value of ten (Fig 2A) and a clear modal value in the distribution of ΔK is detected (Fig 2B). These results suggest the presence of at least ten main groupings among the 142 strains analyzed here.

**Fig 3:**
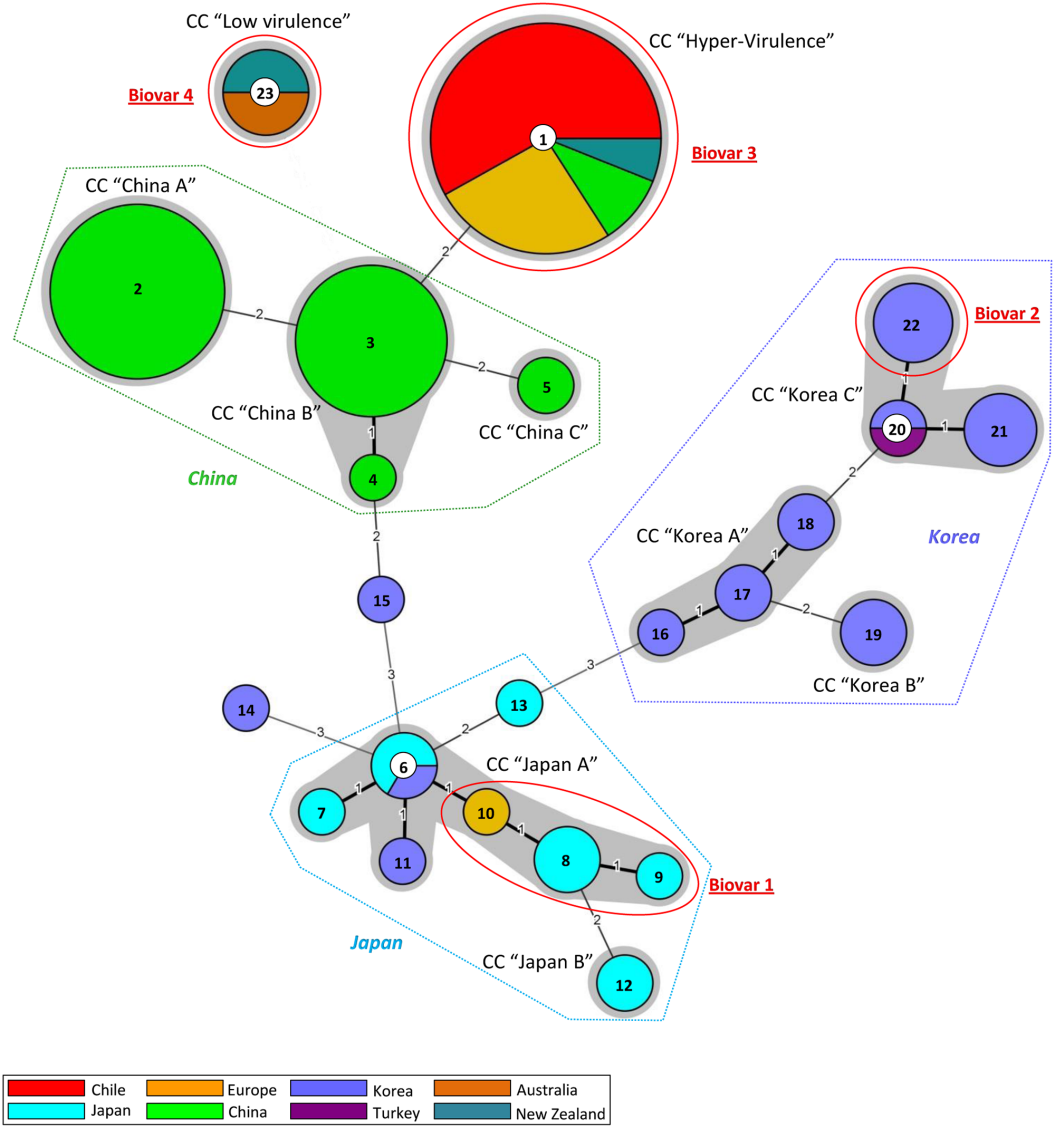
Minimum spanning tree of 142 Psa strains obtained by MLVA6—Panel 1. The MST was obtained by Bionumerics 7.5 analyzing the whole complex of strains on the basis of the six Panel 1 VNTR loci. Each circle represents a group of strains with identical MLVA haplotype and its dimension is proportional to the number of strains included. The grey shaded areas represent the clonal complexes (CC), defined as groups of strains connected with maximum branch length of one. The CCs and singletons circumscribed by dashed lines enclose strains of the main geographical groups: China, Japan and Korea. The red circles enclose the strains that engendered the former definition of 4 Psa biovars.

The result obtained with STRUCTURE analysis is substantially confirmed and described in more details in the following Minimum Spanning Tree (MST) in which, assuming a Clonal Complex (CC) as a group of strains sharing an identical number of repeats on at least five out of six loci, ten distinct clonal complexes and three singletons are recognizable among the 23 different haplotypes (Fig 3).

All the strains representatives of the worldwide epidemic detected in 2008 in Europe, New Zealand and Chile, corresponding to Psa biovar 3, are included into a single clonal complex, named “Hyper-Virulence” together with five Chinese strains isolated in 2010 from two different locations of Shaanxi province. All the fifty strains in this CC share a unique Panel 1 haplotype. This CC is split in numerous haplotypes when all 13 VNTR loci are used (see below)

The remaining Chinese strains are distributed in three related Clonal Complexes. CC “China A” is formed of 28 Chinese strains isolated in 2012: all 26 strains from Guizhou province, Xiuwen county, plus one strain from Shaanxi province, Xi’an district (Haxa 4) and one from Sichuan province, Mianzhu county (Hym 1), have the same Panel 1 haplotype. CC “China B” encompasses 22 strains isolated in 2012 in other different areas of China: twelve from Yuexi county of Anhui province, seven (out of eight) from Xi’an district of Shaanxi province, three (out of four) strains from Mianzhu county of Sichuan province. Twenty-one share an identical Panel 1 haplotype whilst strain JILO30 has one additional repetition (six instead of five) at VNTR locus GM-254. CC “China C” is formed of only two strains with identical haplotype, both isolated during 2010 in Shaanxi province: strain M218, from Yangling county and strain M228, from Mei county. No strain from outside China is included in these three CCs.

Japanese strains are mainly grouped in two CCs. CC “Japan A” encloses seven Psa biovar 1 Japanese strains isolated from Shizuoka prefecture, Chubu region (four in 1984, including the type strain KW11 and three in 2011), one Italian strain isolated in 1992 and two Korean strains, recovered from Naju area (North of Jeollanam province), in 1989. CC “Japan B” includes two strains from CFBP isolated in 1988 from an unknown location in Japan. Among Japanese strains, the strain 2726, isolated also from Shizuoka prefecture but in 2009, clusters as a singleton.

The other Korean strains are divided in three clonal complexes: CC “Korea A” includes all five strains isolated in 2013 in Wando county of Jeollanam province; CC “Korea B” embraces ten Korean strains (seven isolated in different locations of Jeju province and three from Jeollanam province and from 1999 to 2010), plus the only Turkish strain included in the analysis. CC “Korea C” is formed of three strains (SYS1-SYS4) isolated in Goheung county of Jeollanam in 2011. Finally, two Korean strains, K2 and 23665, form two singletons.

The last clonal complex, that shares only one on six VNTR loci with the closest Psa strain, is the CC Psa biovar 4 “Low Virulence”, formed of three strains from New Zealand and three from Australia with a single Panel 1 haplotype.

### Deepening analysis within main populations

The full MLVA-13 (Panel 1 + Panel 2) data of each of those groups were separately submitted to the same clustering analysis reported above and UNJ dendrograms were chosen for the graphical representation of the relationships between strains.

**Fig 4:**
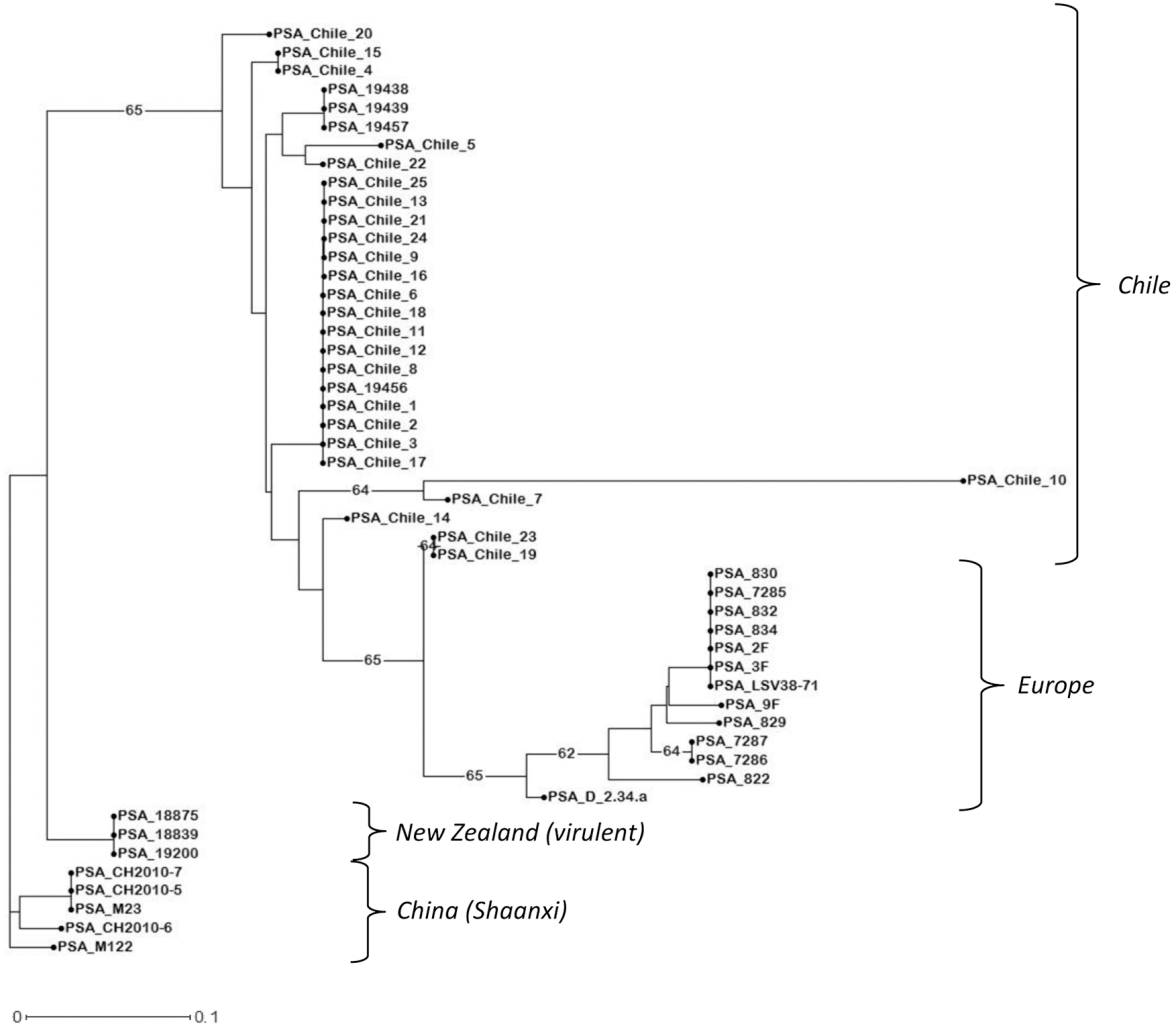
Unweighted Neighbor-Joining dendrogram of the hyper-virulent strains. The UNJ represents results from the analysis of the fifty strains enclosed in the “Hyper-Virulence” group (see Fig 3) with the complete set of 13 VNTR loci. Numbers on branches represents the percentage of bootstraps on 500 replications, when higher than 60%.

The group “Ubiquitous HV” (Fig 4) is constituted by all 50 highly virulent strains linked to the recent global pandemic of Psa. The use of the complete set of 13 VNTR loci allows the distinction of genotypes in agreement with their geographic origin: China, New Zealand, Europe and Chile.

**Fig 5:**
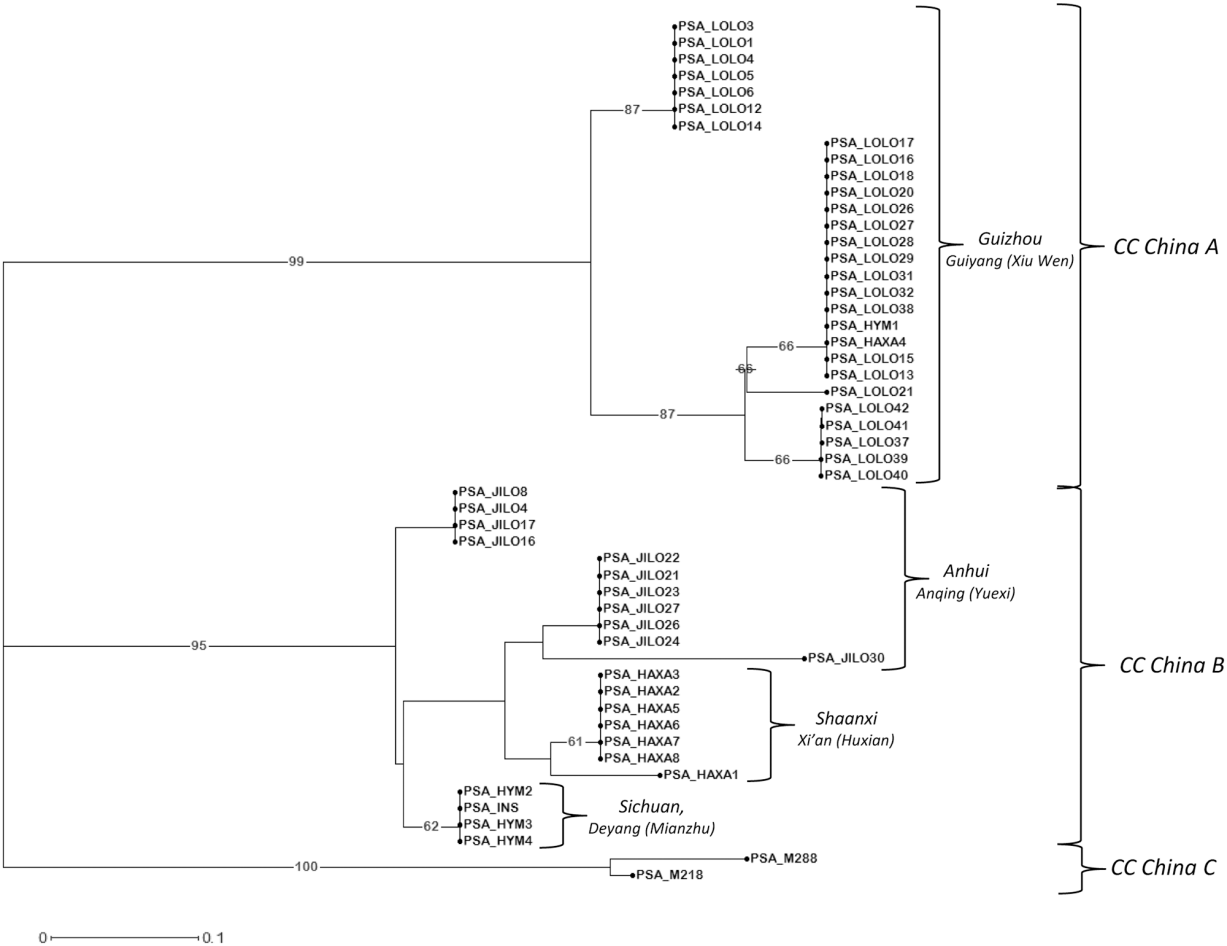
Unweighted Neighbor-Joining dendrogram of the strains from China. The UNJ dendrogram represents results from the analysis of the fifty-two strains enclosed in the “China” group (see Fig 3) with the complete set of 13 VNTR loci. Numbers on branches represents the percentage of bootstraps on 500 replications, when higher than 60%.

The second group is constituted by most Chinese strains (except for the five Chinese strains in the “Hyper-Virulence” group). These fifty-two strains are divided in three main branches of the UNJ dendrogram obtained from the complete dataset analysis, congruently with CCs in the MST based on Panel 1 data, but further groupings are recognizable within (Fig 5). As example, within the branch corresponding to CC “China B”, four subclusters are distinguishable: two contain eleven on twelve strains from Yuexi county of Anhui province; one includes seven on eight strains from Xi’an district, Shaanxi province; the last one includes three on four strains from Mianzhu county of Sichuan province.

**Fig 6:**
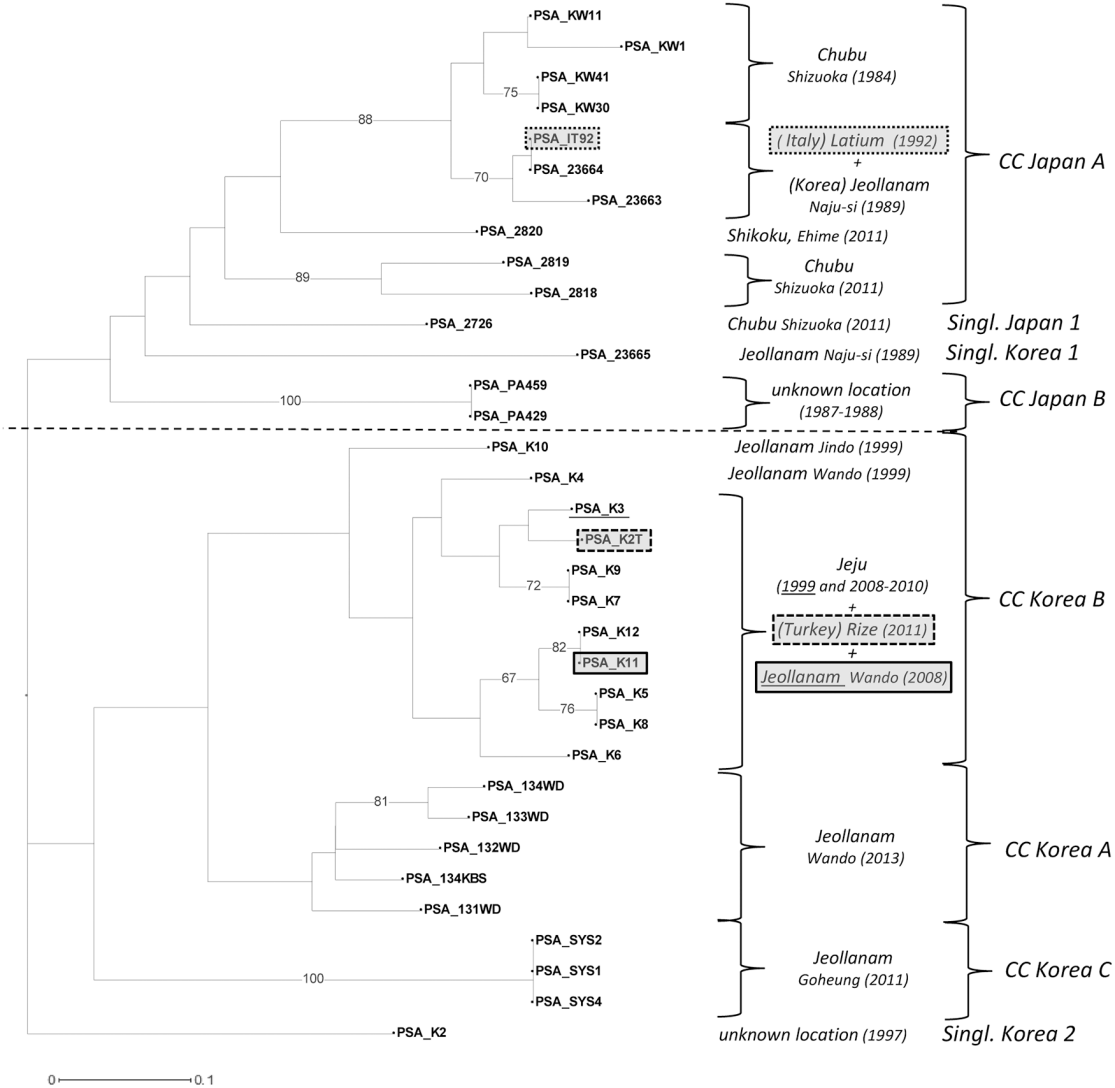
Unweighted Neighbor-Joining dendrogram of the strains from Japan and Korea. The UNJ dendrogram represents results from the analysis of the thirty-four strains enclosed in both the “Japan” and “Korea” groups and related singletons (see Fig 3) with the complete set of 13 VNTR loci. Numbers on branches represents the percentage of bootstraps on 500 replications, when higher than 60%.

The group “Japan-Korea” is the most heterogeneous one. Twenty-six genotypes are resolved by MLVA-13 Panel 1+2 (UNJ dendrogram Fig 6). In the upper part of the dendrogram the branch corresponding to the CC “Japan A” shows a subcluster formed of the four Japanese strains isolated in 1984 in Shizuoka (KW1, KW11, KW30 and KW41) and another one with the Italian strain isolated in 1992 in Latium plus two Korean strains (23663 and 23664) isolated in 1989 in Jeollanam. The Japanese strains isolated in 2011 are weakly clustered, whilst the independent cluster formed of the strains PA429 and PA459 corresponds to the CC “Japan B”.

The lower part of the dendrogram includes almost all the residual Korean strains (16 on 19) splitting them in three clusters, again corresponding to the CCs of Panel 1 analysis. Interestingly, the one corresponding to CC “Korea B” is divided in two subclusters that encompass strains isolated from several locations of Jeollanam and Jeju provinces since 1999 to 2009. The first subcluster also contains the only Turkish strain (K2T) isolated in 2011. The two clusters corresponding to CCs “Korea A” and “Korea B” are still present without significant changes. The Korean strain K2 clusters again as a separate singleton.

In the complete MLVA13 analysis the group “Low Virulence” is even more distant from all the other strains, having as many as ten differences on 13 VNTR loci to the closest strains. The assay distinguishes Australian strains from New Zealand ones due to a one repeat unit difference at the highly variable locus GM-1834.

The data produced was used to create a dedicated resource on the internet by taking advantage of MLVAbank (http://mlva.u-psud.fr). The cooperative *Pseudomonas syringae* pv. *actinidiae* database comprises PSA_in_silico, deduced from *in silico* MLVA typing of WGS data, and PSA_Italia, made from the present MLVA data.

## Discussion

The dangerousness of the bacterial canker of kiwifruit has generated attention from the scientific community on its causal agent *Pseudomonas syringae* pv. *actinidiae*. Since 2008 eighty-three scientific reports were published about this topic in indexed journals (Scopus search), of which twenty-nine in 2014. From an epidemiologic point of view, the main result obtained to date was the definition of 4–5 biovars, of which biovar 3 seems to be the most dangerous. Nevertheless, all these efforts were seriously affected by the scarceness in terms of number and assortment of strains and the use of different analytical methodologies that have made comparison on large scale almost impossible. In this report, conversely, for the first time a wide collection of 142 Psa strains could be used. One hundred and twenty-eight are of recent origin (from 2008 to date) and 14 are “historical strains” (from 1984 to 1999).

We evaluated the MultiLocus VNTR Analysis (MLVA) approach as investigative technique for being simple, cheap, fast, robust, easily reproducible at both intra and inter-laboratory level and properly sensible in detecting small but significant genetic differences. It has become a standard in evaluating the population structure and dynamics of a number of bacterial pathogens, affecting animal or human health [45,71–73]. It is also true for plant pathogenic bacteria, as demonstrated by the increasing number of publications in this field in the recent years (Table 1).

The initial set-up of an efficient and robust MLVA assay is a key step of this technique, greatly facilitated by the availability of genome sequences from strains representative of the species diversity. In the present study, the availability of WGS data of strains having different geographical and temporal origins permitted us to develop a MLVA assay in which 13 VNTR loci have been successfully recognized and used to arrange properly the haplotypes derived from the analysis of a wide collection of Psa strains. If needed, additional VNTRs may be identified, with shorter repeat units and/or overall array length.

Our results are congruent with the distinction in four populations (biovars) outlined in previous studies. We demonstrated that the six VNTR loci, constituting MLVA Panel 1 are sufficient to attain this identification level. Psa biovar 4 is confirmed as quite different from the others, as demonstrated by the very high number of TR differences of corresponding strains with closest relatives, and the lack of amplification of four among the 13 loci.

By this same approach and with the support of STRUCTURE results, it was also suggested that, rather than four, at least ten different populations can be defined within Psa”*sensu latu*”. Whole genome sequence analysis of the corresponding strains will be necessary to confirm this suggestion. The complete set of MLVA data proved also its effectiveness in distinguishing Psa subpopulations and illustrating their correlation mainly with geographical origins but also with isolation time as well as with pathogenicity level. In the present investigation aimed at setting up the assay, monoplex PCRs and capillary electrophoresis were used. In the future, multiplex PCR will obviously be an interesting option. Each panel can easily fit in a single multiplex PCR, as illustrated by earlier works [74,75]. For instance Sobral et al. [74] amplify 12 *Legionella pneumophila* VNTR loci in a single multiplex PCR.

According to previous assessments, Psa from Japan was generically described as Psa biovar1, but this definition refers to the analysis of a handful of historical strains from Shizuoke prefecture plus Italian strains isolated in 1992. Here the existence of this primal core of Psa is confirmed, but its similarity with two Korean strains isolated in 1989 was also shown. Therefore the original Japanese population might have spread during the 80–90’s both in Italy and Korea, where the disease was reported at that time [14,18]. A distinct cluster of Japanese strains isolated in 1987–1988 support the hypothesis that various Psa populations were already present by then. On the other hand, VNTR differences between the original core of Japanese strains and those isolated from the same Shizuoke area in 2011 suggests that different populations nowadays exist in Japan. In support of this hypothesis, Sawada and colleagues [40], according to sequence analysis of ITS, of seven core genome genes and other biochemical and physiological features, reported the presence of a new biovar, named Psa5, whose representatives were isolated from *A*. *chinensis* cv. Hort16A in Saga Prefecture since 2010. The evolution of various Japanese lineages different from the strains reported in the past is also supported by results obtained recently by Ferrante and colleagues [76] using rep-PCR analysis as well as variation in phaseolotoxin gene cluster and type III gene effectors.

Similarly, the data about Korea point to a more complex situation than the few strains generically referred as Psa biovar 2. The haplotypes of strains isolated in 1989 appear more similar to those of ancient Japanese strains than to those of recent strains from Korea, but no evidence of their surviving was found. Our results indicate that at least three distinct populations currently exist. One (CC Korea A), sharply distinct from the others, is residing in Wando county, Jeollanam province. Another one (CC Korea C) was recovered in Goheung county of the same province. This is formed of the same strains analyzed by Koh and colleagues [16,17] which, based on sequencing six housekeeping genes and three effector genes, conclude that these represent a new type of Psa, somehow referable to the Italian contemporary population. This study does not support any association with Italian haplotypes.

Koh and colleagues [15] reported a virulent outbreak of bacterial canker in Jeju province since 2006, particularly harmful on *A*. *chinensis*. This population could correspond to our third Korean group (CC Korea B) of which seven (out of ten) strains were isolated in Jeju province in those years.

Relevant to the prospect of disease diffusion, the unique Turkish strain available and related to the report of Bastas and Karakaya [25] has an haplotypevery similar to this new population and calls into question whether this could be the first case of international spread of this lineage.

In the last years higher attention was paid on the hyper-virulent population of Psa biovar 3. Since the first WGS investigations of Psa, a highly clonal structure of this aggressive population was depicted and a close relationship between Psa3 and few Chinese strains isolated from Shaanxi region in 2010 was reported. Even the most recent report about Psa population in Shaanxi Province [13], analyzing 16S rDNA sequences and rep-PCR fingerprinting of twelve strains collected on seven kiwifruit species and cultivars in 2010–2011, did not find substantial differences with current strains from Italy or New Zealand. Both the high clonality of Psa biovar 3 (all the strains belong to a single MLVA CC), and its similarity with a group of Chinese strains (the five strains from Dangdong and Mei areas in Shaanxi province included in the same MLVA CC) are well represented in our analysis. Furthermore the complete MLVA assay demonstrated to be able to group hypervirulent strains according to their specific geographical origin.

Concerning the “low virulent” Psa biovar 4, this MLVA assay and all the previous molecular researches merge into the conclusion that it is a separate group, with its own genetic features, to the extent that it was included in the new pathovar *Pseudomonas syringae* pv. *actinidifoliorum* [39]. Interestingly, the six strains investigated here, three from New Zealand and three from Australia, are very closely related. This is in agreement with the previous report by Cunty et al suggesting a higher genetic diversity of biovar 4 in Europe compared to New Zealand and Australia.

One of the most important novelty of this study concerns the analysis of Psa populations in China. Being the country of origin of *Actinidia* genus and a place where the disease has been reported for a long time, presumably a time of coevolution of host and pathogen in China was initiated before cultivation in other countries. It was consequently not unexpected that China turned out to be the country where the widest variability resides, even if it had not been demonstrated before.

Our results show indeed that strains from distinct kiwifruit cultivation areas of China, all isolated after 2010, constitute, or are part of, at least four different groupings. Among them, the one represented by CC “China B” has the widest geographical dissemination in China, including groups of strains isolated in three areas (Anhui, Shaanxi, and Sichuan provinces) thousands of kilometers apart. Another well-defined and separate population resides in the Xiu Wen county, in Anhui province, as represented by the clonal complex “China A”. This complex also includes one strain from Xi’an and one from Mianzhu counties in this group, suggesting diffusion of this haplotype. Besides the five strains from Shaanxi included in the hypervirulent clonal complex, the last CC “China C” is also composed by two strains from the same province, representing an additional distinct population.

The present results show that fifteen strains obtained from Shaanxi province, in a radius of only about 50 kilometers, belong to as many as three different populations. Such significant variability can probably be explained considering that Shaanxi has China’s largest kiwifruit cultivated area and is ascribed for a third of the global kiwifruit production (http://new.shaanxiinvest.gov.cn/tzdt/show.asp?id=3991) of China. In addition, most of the companies producing and commercializing kiwifruit, pollen and vegetative materials for both internal and external markets are located in Shaanxi.

Even though the pathogenicity of Asiatic strains (Chinese, Korean and Japanese) has not been comparatively evaluated yet, it is plausible that this massive amount of diversity would mirror a reservoir of (pathogenicity) genes worth monitoring.

Nowadays the key means of spread of this pathogen are recognized. Visually healthy fruits are considered free of contamination risk [77,78], whilst a significant risk is blamed to pollen [79–81], subject to national and international marketing for its role in the amelioration of fruit size and shape by artificial pollination. But the highest risk is surely related to the commerce of contaminated vegetative material, because of the well-known Psa colonization of the vascular system of kiwifruit plants without producing early symptoms [82,83].

In such sense this MLVA assay has a great potential as easy and effective tool not only to recognize and schedule the presence of different Psa types all over the world, but above all to trace their movements on local to international scale, supporting the simple detection of contaminated materials with key information concerning specific haplotypes. It will be interesting to evaluate if a first-line assay based upon one multiplex PCR assay for MLVA typing can be applied to raw DNA material.

In this study MLVA has confirmed to be more sensible than many others classical molecular analysis with comparable running costs. From a technical point of view, the incongruities reported between WGS and true VNTR sequences demonstrated that tandem repeats are poorly analyzable with short reads WGS sequencing. However, this problem is progressively being solved by the availability of much longer reads [84].

Finally, the chance to generate a haplotypes database accessible via the internet (MLVAbank), in a way which makes simple similarity queries very straightforward and user-friendly for worldwide research community makes MLVA even more promising. This could be of even greater importance considering that the assay presented here is not a closed system. It can be seen as a starting point that could be improved, by the addition of new VNTR loci effective in resolving the complex evolution of a pathogen such as Psa. In this view, MLVA would be used as first-line assay, followed by WGS analysis of relevant samples.

## Supporting Information

S1 Table List of the 142 *Pseudomonas syringae pv*. *actinidiae* strains used in this study. Additional information about sources, year of isolation and detailed geographical origin of each strain are reported. In the case of Chinese strains the county of origin is indicated in brackets in the locality column. MLVA genotypes of the strains are also included.
(DOC)
