# Supplementary material for: Development of a Multiple Loci Variable Number of Tandem Repeats Analysis (MLVA) to Unravel the Intra-Pathovar Structure of Pseudomonas syringae pv. actinidiae Populations Worldwide
Source: PLoS One. 2015 Aug 11;10(8):e0135310. doi: 10.1371/journal.pone.0135310 (PMC4532359; doi:10.1371/journal.pone.0135310)
Supplement: S1 Table — Additional information about sources, year of isolation and detailed geographical origin of each strain are reported. In the case of Chinese strains the county of origin is indicated in brackets in the locality column. MLVA genotypes of the strains are also included. (DOC) [file pone.0135310.s001.doc]

| **Strain code** | **Source** | **Year** | **Country** | **Region** | **Locality** | **Host species** | **Cultivar** | **Psa-01** | **Psa-03** | **Psa-04** | **Psa-05** | **Psa-06** | **Psa-07** | **Psa-08** | **Psa-09** | **Psa-10** | **GM-254** | **GM-1553** | **GM-1834** | **GM-4076** |
| --- | --- | --- | --- | --- | --- | --- | --- | --- | --- | --- | --- | --- | --- | --- | --- | --- | --- | --- | --- | --- |
| *In silico* ref. strain  ICMP 18884  GenBank Acc. n°  NZ_CM002752.1 | ICMP | 2010 | New Zealand | Bay of Plenty | Te Puke | *A. deliciosa* | Hayward | 9 | 10 | 3 | 6 | 4 | 2 | 3 | 6 | 14 | 5 | 6 | 18 | 2 |
| PSA_7285 | CFBP | 2008 | Italy | Veneto | Treviso | *A. chinensis* | Jin Tao | 9 | 10 | 3 | 6 | 4 | 2 | 3 | 6 | 6 | 5 | 6 | 18 | 2 |
| PSA_7286*^†^ | CFBP | 2008 | Italy | Latium | Latina | *A. chinensis* | Hort-16A | 9 | 10 | 3 | 6 | 4 | 2 | 3 | 6 | 6 | 5 | 6 | 17 | 2 |
| PSA_7287 | CFBP | 2008 | Italy | Latium | Latina | *A. deliciosa* | Hayward | 9 | 10 | 3 | 6 | 4 | 2 | 3 | 6 | 6 | 5 | 6 | 17 | 2 |
| PSA_829 | UNITUS | 2011 | Spain | Galicia | Borguiera Tomino | *A. chinensis* | Jin Tao | 9 | 10 | 3 | 5 | 4 | 2 | 3 | 6 | 6 | 5 | 6 | 18 | 2 |
| PSA_830^†^ | UNITUS | 2011 | Spain | Galicia | Borguiera Tomino | *A. chinensis* | Jin Tao | 9 | 10 | 3 | 6 | 4 | 2 | 3 | 6 | 6 | 5 | 6 | 18 | 2 |
| PSA_822^†^ | UNITUS | 2011 | Portugal | Viana do Castelo | Valença | *A. deliciosa* | Bo.Erica | 9 | 11 | 3 | 6 | 4 | 2 | 3 | 6 | 6 | 5 | 6 | 16 | 2 |
| PSA_832 | UNITUS | 2011 | Portugal | Aveiro | S.ta Maria da Feira | *A. deliciosa* | Hayward | 9 | 10 | 3 | 6 | 4 | 2 | 3 | 6 | 6 | 5 | 6 | 18 | 2 |
| PSA_834 | UNITUS | 2011 | Portugal | Aveiro | S.ta Maria da Feira | *A. deliciosa* | Hayward | 9 | 10 | 3 | 6 | 4 | 2 | 3 | 6 | 6 | 5 | 6 | 18 | 2 |
| PSA_2F^†^ | Anses | 2010 | France | Rhone Alpes | - | *A. deliciosa* | Hayward | 9 | 10 | 3 | 6 | 4 | 2 | 3 | 6 | 6 | 5 | 6 | 18 | 2 |
| PSA_3F | Anses | 2010 | France | Rhone Alpes | - | *A. deliciosa* | Hayward | 9 | 10 | 3 | 6 | 4 | 2 | 3 | 6 | 6 | 5 | 6 | 18 | 2 |
| PSA_9F | Anses | 2011 | France | Rhone Alpes | - | Actinidia sp. | unknown | 9 | 11 | 3 | 6 | 4 | 2 | 3 | 6 | 6 | 5 | 6 | 18 | 2 |
| PSA_LSV38-71 | Anses | 2011 | Switzerland | Canton of Geneva | Meynier | *A. deliciosa* | Chico | 9 | 10 | 3 | 6 | 4 | 2 | 3 | 6 | 6 | 5 | 6 | 18 | 2 |
| PSA_D_2.34a | LTZ-Aug. | 2013 | Germany | Bavaria | - | *A. chinensis* | Sweet-Heart | 9 | 10 | 3 | 6 | 4 | 2 | 3 | 6 | 8 | 5 | 6 | 18 | 2 |
| PSA_IT92* | CRA-FRU | 1992 | Italy | Latium | Latina | *A. deliciosa* | Hayward | 3 | 3 | - | 4 | 3 | 3 | 10 | 7 | 14 | 12 | 4 | 15 | 3 |
| PSA_K2T^†^ | Bastas K.K. | 2011 | Turkey | Rize | - | *A. deliciosa* | Hayward | 6 | 4 | 2 | 6 | 4 | 3 | 3 | 6 | 12 | 8 | 5 | 12 | 4 |
| PSA_18804* | ICMP | 2010 | New Zealand | Bay of Plenty | Te Puke | *A. chinensis* | Hort 16A | 3 | 3 | 1 | 2 | ***-*** | ***-*** | 2 | ***-*** | 2 | ***-*** | 3 | 7 | 2 |
| PSA­_18882 | ICMP | 2010 | New Zealand | Nelson | Motueka | *A. chinensis* | Hort 16A | 3 | 3 | 1 | 2 | ***-*** | ***-*** | 2 | ***-*** | 2 | ***-*** | 3 | 7 | 2 |
| PSA_19497 | ICMP | 2010 | New Zealand | Bay of Plenty | Te Puke | *A. chinensis* | Hort 16A | 3 | 3 | 1 | 2 | ***-*** | ***-*** | 2 | ***-*** | 2 | ***-*** | 3 | 7 | 2 |
| PSA_19441 | ICMP | 2012 | Australia | Victoria | - | *A. chinensis* | unknown | 3 | 3 | 1 | 2 | ***-*** | ***-*** | 2 | ***-*** | 2 | ***-*** | 3 | 8 | 2 |
| PSA_19486 | ICMP | 2013 | Australia | Western Australia | - | *A. chinensis* | unknown | 3 | 3 | 1 | 2 | ***-*** | ***-*** | 2 | ***-*** | 2 | ***-*** | 3 | 8 | 2 |
| PSA_19440 | ICMP | 2011 | Australia | Victoria | - | *A. chinensis* | unknown | 3 | 3 | 1 | 2 | ***-*** | ***-*** | 2 | ***-*** | 2 | ***-*** | 3 | 8 | 2 |
| PSA_18839 | ICMP | 2011 | New Zealand | Bay of Plenty | Te Puke | *A. deliciosa* | Hayward | 9 | 10 | 3 | 6 | 4 | 2 | 3 | 6 | 14 | 5 | 6 | 18 | 2 |
| PSA_18875^†^ | ICMP | 2011 | New Zealand | Bay of Plenty | Te Puke | *A. deliciosa* | Hayward | 9 | 10 | 3 | 6 | 4 | 2 | 3 | 6 | 14 | 5 | 6 | 18 | 2 |
| PSA_19200 | ICMP | 2011 | New Zealand | Auckland | - | Actinidia sp. | unknown | 9 | 10 | 3 | 6 | 4 | 2 | 3 | 6 | 14 | 5 | 6 | 18 | 2 |
| PSA_23663 | KCTC | 1989 | Korea | Jeollanam | Naju-si | *A. chinensis* | unknown | 3 | 3 | 3 | 4 | 3 | 3 | 10 | 7 | 14 | 12 | 6 | 15 | 3 |
| PSA_23664^†^ | KCTC | 1989 | Korea | Jeollanam | Naju-si | *A. chinensis* | unknown | 3 | 3 | 3 | 4 | 3 | 3 | 10 | 7 | 14 | 12 | 4 | 15 | 3 |
| PSA_23665 | KCTC | 1989 | Korea | Jeollanam | Naju-si | *A. chinensis* | unknown | 5 | 6 | 3 | 8 | 3 | 3 | 10 | 3 | 7 | 6 | 8 | 10 | 3 |
| PSA_K2^†^ | Koh Y.J. | 1997 | Korea | - | - | *A. chinensis* | unknown | 3 | 6 | 3 | 7 | 4 | 4 | 4 | 7 | 10 | 8 | 4 | 6 | 4 |
| PSA­_K3 | Koh Y.J | 1999 | Korea | Jeju | Bukjeju-gun | *A. deliciosa* | Hayward | 5 | 4 | 2 | 6 | 4 | 3 | 3 | ***-*** | 12 | 8 | 5 | 12 | 4 |
| PSA_K4 | Koh Y.J | 1999 | Korea | Jeollanam | Wando-gun | *A. deliciosa* | Hayward | 5 | 4 | 2 | 6 | 4 | 3 | 3 | ***-*** | 10 | 8 | 5 | 13 | 4 |
| PSA_K5 | Koh Y.J | 2008 | Korea | Jeju | Nabeup-ri | *A. deliciosa* | Hayward | 6 | 4 | 2 | 6 | 4 | 3 | 3 | ***-*** | 11 | 7 | 5 | 17 | 4 |
| PSA_K6 | Koh Y.J | 2010 | Korea | Jeju | Tosan-ri | *A. chinensis* | Hort-16A | 6 | 4 | 2 | 6 | 4 | 3 | 3 | ***-*** | 12 | 8 | 5 | 17 | 4 |
| PSA_K7 | Koh Y.J | 2008 | Korea | Jeju | Tosan-ri | *A. chinensis* | Hort-16A | 5 | 4 | 2 | 6 | 4 | 3 | 3 | ***-*** | 11 | 8 | 5 | 12 | 4 |
| PSA_K8 | Koh Y.J | 2008 | Korea | Jeju | Sangmo-ri | *A. chinensis* | Hort-16A | 6 | 4 | 2 | 6 | 4 | 3 | 3 | ***-*** | 11 | 7 | 5 | 17 | 4 |
| PSA_K9 | Koh Y.J | 2008 | Korea | Jeju | Odeung-dong | *A. chinensis* | Hort-16A | 5 | 4 | 2 | 6 | 4 | 3 | 3 | ***-*** | 11 | 8 | 5 | 12 | 4 |
| PSA_K10 | Koh Y.J | 1999 | Korea | Jeollanam | Jindo-gun | *A. deliciosa* | Hayward | 5 | 4 | 2 | 6 | 3 | 3 | 3 | ***-*** | 10 | 8 | 5 | 15 | 4 |
| PSA_K11 | Koh Y.J | 2008 | Korea | Jeollanam | Wando-gun | *A. deliciosa* | Hayward | 6 | 4 | 2 | 6 | 4 | 3 | 3 | ***-*** | 10 | 7 | 5 | 17 | 4 |
| PSA_K12 | Koh Y.J | 2009 | Korea | Jeju | Tosan-ri | *A. chinensis* | Hort-16A | 6 | 4 | 2 | 6 | 4 | 3 | 3 | ***-*** | 10 | 7 | 5 | 17 | 4 |
| PSA_131WD | Koh Y.J. | 2013 | Korea | Jeollanam | Wando-eup | *A. chinensis* | Hong Yang | 7 | 4 | 2 | 7 | 4 | 3 | 3 | ***-*** | 10 | 8 | 4 | 13 | 4 |
| PSA_132WD | Koh Y.J. | 2013 | Korea | Jeollanam | Wando-eup | *A. chinensis* | Hong Yang | 7 | 4 | 2 | 7 | 4 | 3 | 3 | ***-*** | 10 | 5 | 4 | 13 | 4 |
| PSA_133WD | Koh Y.J. | 2013 | Korea | Jeollanam | Wando-eup | *A. chinensis* | new variety | 6 | 4 | 2 | 7 | 3 | 3 | 2 | ***-*** | 10 | 5 | 4 | 13 | 4 |
| PSA­_134WD | Koh Y.J. | 2013 | Korea | Jeollanam | Wando-eup | *A. chinensis* | new variety | 7 | 4 | 2 | 7 | 4 | 3 | 2 | ***-*** | 10 | 5 | 4 | 13 | 4 |
| PSA_134KBS | Koh Y.J. | 2013 | Korea | Jeollanam | Yeongam-eup | *A. deliciosa* | Hayward | 6 | 4 | 2 | 7 | 3 | 3 | 3 | ***-*** | 10 | 5 | 4 | 13 | 4 |
| PSA_SYS1 | Koh Y.J. | 2011 | Korea | Jeollanam | Goheung-gun | *A. chinensis* | unknown | 5 | 7 | 2 | 7 | 2 | 3 | 8 | ***3*** | 6 | 5 | 4 | 11 | 2 |
| PSA_SYS2 | Koh Y.J. | 2011 | Korea | Jeollanam | Goheung-gun | *A. chinensis* | unknown | 5 | 7 | 2 | 7 | 2 | 3 | 8 | ***3*** | 6 | 5 | 4 | 11 | 2 |
| PSA_SYS4 | Koh Y.J. | 2011 | Korea | Jeollanam | Goheung-gun | *A. chinensis* | unknown | 5 | 7 | 2 | 7 | 2 | 3 | 8 | ***3*** | 6 | 5 | 4 | 11 | 2 |
| PSA_KW1* | Takikawa Y. | 1984 | Japan | Chūbu | Shizuoka | *A. deliciosa* | Hayward | 4 | 3 | ***-*** | 4 | 3 | 4 | 10 | 7 | 14 | 12 | 4 | 15 | 3 |
| PSA_KW11* | Takikawa Y. | 1984 | Japan | Chūbu | Shizuoka | *A. deliciosa* | Hayward | 4 | 3 | ***-*** | 4 | 3 | 3 | 10 | 7 | 14 | 12 | 4 | 15 | 3 |
| PSA_KW30 | CFBP | 1984 | Japan | Chūbu | Shizuoka | *A. deliciosa* | Hayward | 4 | 3 | ***-*** | 4 | 3 | 3 | 10 | 7 | 14 | 12 | 4 | 14 | 3 |
| PSA_KW41* | CFBP | 1984 | Japan | Chūbu | Shizuoka | *A. deliciosa* | Hayward | 4 | 3 | ***-*** | 4 | 3 | 3 | 10 | 7 | 14 | 12 | 4 | 14 | 3 |
| PSA_PA429 | CFBP | 1987 | Japan | - | - | *A. chinensis* | unknown | 4 | 4 | ***-*** | 5 | 3 | 2 | 4 | 7 | 16 | 12 | 4 | 14 | 4 |
| PSA_PA459* | CFBP | 1988 | Japan | - | - | *A. chinensis* | unknown | 4 | 4 | ***-*** | 5 | 3 | 2 | 4 | 7 | 16 | 12 | 4 | 14 | 4 |
| PSA­_2818 | Takikawa Y. | 2011 | Japan | Chūbu | Shizuoka | *A. chinensis* | Rainbow red | 3 | 3 | 3 | 6 | 6 | 3 | 8 | 7 | 17 | 11 | 4 | 11 | 3 |
| PSA_2819 | Takikawa Y. | 2011 | Japan | Chūbu | Shizuoka | *A. chinensis* | Rainbow red | 3 | 3 | 3 | 6 | 6 | 3 | 9 | 7 | 16 | 12 | 4 | 11 | 3 |
| PSA_2820 | Takikawa Y. | 2011 | Japan | Shikoku | Ehime | *A. deliciosa* | Hayward | 3 | 4 | 3 | 4 | 3 | 3 | 4 | 7 | 13 | 12 | 4 | 9 | 3 |
| PSA_2726 | Takikawa Y. | 2009 | Japan | Chūbu | Shizuoka | *A. deliciosa* | unknown | 4 | 3 | 3 | 7 | 4 | 3 | 10 | 7 | 11 | 8 | 4 | 7 | 3 |
| PSA_CH2010-5 | Huang L.L. | 2010 | China | Shaanxi | Sheng (Dangdong) | *A. chinensis* | Hong Yang | 9 | 11 | 3 | 5 | 4 | 2 | 4 | 6 | 14 | 5 | 6 | 18 | 2 |
| PSA_CH2010-6*^†^ | Huang L.L. | 2010 | China | Shaanxi | Sheng (Dangdong) | *A. chinensis* | Hong Yang | 9 | 11 | 3 | 5 | 4 | 2 | 4 | 6 | 14 | 5 | 6 | 18 | 2 |
| PSA_CH2010-7 | Huang L.L. | 2010 | China | Shaanxi | Sheng (Dangdong) | *A. chinensis* | Hong Yang | 9 | 11 | 3 | 5 | 4 | 2 | 4 | 6 | 14 | 5 | 6 | 18 | 2 |
| PSA_M218 | Zhao Z.B. | 2010 | China | Shaanxi | Xianyang (Yangling) | *A. deliciosa* | Xi Xuan | 5 | 7 | 3 | 6 | 4 | 2 | 9 | 7 | 9 | 4 | 4 | 16 | 2 |
| PSA_M228 | Zhao Z.B. | 2010 | China | Shaanxi | Baoji (Mei) | *A. chinensis* | Hong Yang | 5 | 7 | 3 | 6 | 5 | 2 | 9 | 7 | 9 | 4 | 4 | 16 | 2 |
| PSA­_M23 | Zhao Z.B. | 2010 | China | Shaanxi | Baoji (Mei) | *A. chinensis* | Hong Yang | 9 | 11 | 3 | 5 | 4 | 2 | 4 | 6 | 14 | 5 | 6 | 18 | 2 |
| PSA­_M122 | Zhao Z.B. | 2010 | China | Shaanxi | Baoji (Mei) | *A. chinensis* | Hong Yang | 9 | 11 | 3 | 5 | 4 | 2 | 4 | 6 | 14 | 5 | 6 | 18 | 2 |
| PSA_JILO4^†^ | UNITUS | 2012 | China | Anhui | Anqing (Yuexi) | *A. chinensis* | JinFeng | 5 | 10 | 3 | 7 | 4 | 2 | 7 | 7 | 12 | 5 | 8 | 8 | 2 |
| PSA_JILO8 | UNITUS | 2012 | China | Anhui | Anqing (Yuexi) | *A. chinensis* | JinFeng | 5 | 10 | 3 | 7 | 4 | 2 | 7 | 7 | 12 | 5 | 8 | 8 | 2 |
| PSA_JILO16 | UNITUS | 2012 | China | Anhui | Anqing (Yuexi) | *A. chinensis* | JinFeng | 5 | 10 | 3 | 7 | 4 | 2 | 7 | 7 | 12 | 5 | 8 | 8 | 2 |
| PSA_JILO17 | UNITUS | 2012 | China | Anhui | Anqing (Yuexi) | *A. chinensis* | JinFeng | 5 | 10 | 3 | 7 | 4 | 2 | 7 | 7 | 12 | 5 | 8 | 8 | 2 |
| PSA_JILO21 | UNITUS | 2012 | China | Anhui | Anqing (Yuexi) | *A. chinensis* | JinFeng | 5 | 10 | 3 | 7 | 3 | 2 | 7 | 7 | 12 | 5 | 8 | 8 | 2 |
| PSA_JILO22 | UNITUS | 2012 | China | Anhui | Anqing (Yuexi) | *A. chinensis* | JinFeng | 5 | 10 | 3 | 7 | 3 | 2 | 7 | 7 | 12 | 5 | 8 | 8 | 2 |
| PSA_JILO23 | UNITUS | 2012 | China | Anhui | Anqing (Yuexi) | *A. chinensis* | JinFeng | 5 | 10 | 3 | 7 | 3 | 2 | 7 | 7 | 12 | 5 | 8 | 8 | 2 |
| PSA_JILO24 | UNITUS | 2012 | China | Anhui | Anqing (Yuexi) | *A. chinensis* | JinFeng | 5 | 10 | 3 | 7 | 3 | 2 | 7 | 7 | 12 | 5 | 8 | 8 | 2 |
| PSA_JILO26 | UNITUS | 2012 | China | Anhui | Anqing (Yuexi) | *A. chinensis* | JinFeng | 5 | 10 | 3 | 7 | 3 | 2 | 7 | 7 | 12 | 5 | 8 | 8 | 2 |
| PSA_JILO27 | UNITUS | 2012 | China | Anhui | Anqing (Yuexi) | *A. chinensis* | JinFeng | 5 | 10 | 3 | 7 | 3 | 2 | 7 | 7 | 12 | 5 | 8 | 8 | 2 |
| PSA_JILO30 | UNITUS | 2012 | China | Anhui | Anqing (Yuexi) | *A. chinensis* | JinFeng | 5 | 10 | 3 | 8 | 3 | 2 | 7 | 7 | 12 | 6 | 8 | 8 | 2 |
| PSA_LOLO1 | UNITUS | 2012 | China | Guizhou | Guiyang (Xiuwen) | *A. deliciosa* | Guichang | 6 | 10 | 3 | 4 | 4 | 2 | 4 | 6 | 16 | 5 | 5 | 16 | 2 |
| PSA_LOLO3 | UNITUS | 2012 | China | Guizhou | Guiyang (Xiuwen) | *A. deliciosa* | Guichang | 6 | 10 | 3 | 4 | 4 | 2 | 4 | 6 | 16 | 5 | 5 | 16 | 2 |
| PSA_LOLO4 | UNITUS | 2012 | China | Guizhou | Guiyang (Xiuwen) | *A. deliciosa* | Guichang | 6 | 10 | 3 | 4 | 4 | 2 | 4 | 6 | 16 | 5 | 5 | 16 | 2 |
| PSA_LOLO5 | UNITUS | 2012 | China | Guizhou | Guiyang (Xiuwen) | *A. deliciosa* | Guichang | 6 | 10 | 3 | 4 | 4 | 2 | 4 | 6 | 16 | 5 | 5 | 16 | 2 |
| PSA_LOLO6 | UNITUS | 2012 | China | Guizhou | Guiyang (Xiuwen) | *A. deliciosa* | Guichang | 6 | 10 | 3 | 4 | 4 | 2 | 4 | 6 | 16 | 5 | 5 | 16 | 2 |
| PSA_LOLO12 | UNITUS | 2012 | China | Guizhou | Guiyang (Xiuwen) | *A. deliciosa* | Guichang | 6 | 10 | 3 | 4 | 4 | 2 | 4 | 6 | 16 | 5 | 5 | 16 | 2 |
| PSA_LOLO13 | UNITUS | 2012 | China | Guizhou | Guiyang (Xiuwen) | *A. deliciosa* | Guichang | 6 | 10 | 3 | 4 | 4 | 2 | 4 | 6 | 16 | 5 | 5 | 16 | 2 |
| PSA_LOLO14 | UNITUS | 2012 | China | Guizhou | Guiyang (Xiuwen) | *A. deliciosa* | Guichang | 6 | 12 | 3 | 4 | 4 | 2 | 4 | 6 | 16 | 5 | 5 | 15 | 2 |
| PSA_LOLO15 | UNITUS | 2012 | China | Guizhou | Guiyang (Xiuwen) | *A. deliciosa* | Guichang | 6 | 12 | 3 | 4 | 4 | 2 | 4 | 6 | 16 | 5 | 5 | 15 | 2 |
| PSA_LOLO16 | UNITUS | 2012 | China | Guizhou | Guiyang (Xiuwen) | *A. deliciosa* | Guichang | 6 | 12 | 3 | 4 | 4 | 2 | 4 | 6 | 16 | 5 | 5 | 15 | 2 |
| PSA_LOLO17 | UNITUS | 2012 | China | Guizhou | Guiyang (Xiuwen) | *A. deliciosa* | Guichang | 6 | 12 | 3 | 4 | 4 | 2 | 4 | 6 | 16 | 5 | 5 | 15 | 2 |
| PSA_LOLO18 | UNITUS | 2012 | China | Guizhou | Guiyang (Xiuwen) | *A. deliciosa* | Guichang | 6 | 12 | 3 | 4 | 4 | 2 | 4 | 6 | 16 | 5 | 5 | 15 | 2 |
| PSA_LOLO20 | UNITUS | 2012 | China | Guizhou | Guiyang (Xiuwen) | *A. deliciosa* | Guichang | 6 | 12 | 3 | 4 | 4 | 2 | 4 | 6 | 16 | 5 | 5 | 15 | 2 |
| PSA_LOLO21 | UNITUS | 2012 | China | Guizhou | Guiyang (Xiuwen) | *A. deliciosa* | Guichang | 6 | 13 | 3 | 4 | 4 | 2 | 4 | 6 | 16 | 5 | 5 | 15 | 2 |
| PSA_LOLO26 | UNITUS | 2012 | China | Guizhou | Guiyang (Xiuwen) | *A. deliciosa* | Guichang | 6 | 12 | 3 | 4 | 4 | 2 | 4 | 6 | 16 | 5 | 5 | 15 | 2 |
| PSA_LOLO27 | UNITUS | 2012 | China | Guizhou | Guiyang (Xiuwen) | *A. deliciosa* | Guichang | 6 | 12 | 3 | 4 | 4 | 2 | 4 | 6 | 16 | 5 | 5 | 15 | 2 |
| PSA_LOLO28 | UNITUS | 2012 | China | Guizhou | Guiyang (Xiuwen) | *A. deliciosa* | Guichang | 6 | 12 | 3 | 4 | 4 | 2 | 4 | 6 | 16 | 5 | 5 | 15 | 2 |
| PSA_LOLO29 | UNITUS | 2012 | China | Guizhou | Guiyang (Xiuwen) | *A. deliciosa* | Guichang | 6 | 12 | 3 | 4 | 4 | 2 | 4 | 6 | 16 | 5 | 5 | 15 | 2 |
| PSA_LOLO31 | UNITUS | 2012 | China | Guizhou | Guiyang (Xiuwen) | *A. deliciosa* | Guichang | 6 | 12 | 3 | 4 | 4 | 2 | 4 | 6 | 16 | 5 | 5 | 15 | 2 |
| PSA_LOLO32 | UNITUS | 2012 | China | Guizhou | Guiyang (Xiuwen) | *A. deliciosa* | Guichang | 6 | 12 | 3 | 4 | 4 | 2 | 4 | 6 | 16 | 5 | 5 | 15 | 2 |
| PSA_LOLO37 | UNITUS | 2012 | China | Guizhou | Guiyang (Xiuwen) | *A. deliciosa* | Guichang | 6 | 11 | 3 | 4 | 4 | 2 | 4 | 6 | 16 | 5 | 5 | 15 | 2 |
| PSA_LOLO38 | UNITUS | 2012 | China | Guizhou | Guiyang (Xiuwen) | *A. deliciosa* | Guichang | 6 | 12 | 3 | 4 | 4 | 2 | 4 | 6 | 16 | 5 | 5 | 15 | 2 |
| PSA_LOLO39 | UNITUS | 2012 | China | Guizhou | Guiyang (Xiuwen) | *A. deliciosa* | Guichang | 6 | 11 | 3 | 4 | 4 | 2 | 4 | 6 | 16 | 5 | 5 | 15 | 2 |
| PSA_LOLO40 | UNITUS | 2012 | China | Guizhou | Guiyang (Xiuwen) | *A. deliciosa* | Guichang | 6 | 11 | 3 | 4 | 4 | 2 | 4 | 6 | 16 | 5 | 5 | 15 | 2 |
| PSA_LOLO41 | UNITUS | 2012 | China | Guizhou | Guiyang (Xiuwen) | *A. deliciosa* | Guichang | 6 | 11 | 3 | 4 | 4 | 2 | 4 | 6 | 16 | 5 | 5 | 15 | 2 |
| PSA_LOLO42 | UNITUS | 2012 | China | Guizhou | Guiyang (Xiuwen) | *A. deliciosa* | Guichang | 6 | 11 | 3 | 4 | 4 | 2 | 4 | 6 | 16 | 5 | 5 | 15 | 2 |
| PSA_INS | UNITUS | 2012 | China | Anhui | Anqing (Yuexi) | Insect (C*icadellidae*) | | 5 | 10 | 3 | 7 | 4 | 2 | 6 | 7 | 12 | 5 | 8 | 8 | 2 |
| PSA_HAXA1 | UNITUS | 2012 | China | Shaanxi | Xi’an (Huxian) | *A. deliciosa* | Hayward | 5 | 11 | 3 | 7 | 3 | 2 | 6 | 7 | 12 | 5 | 8 | 8 | 2 |
| PSA_HAXA2 | UNITUS | 2012 | China | Shaanxi | Xi’an (Huxian) | *A. deliciosa* | Hayward | 5 | 10 | 3 | 7 | 3 | 2 | 6 | 7 | 12 | 5 | 8 | 8 | 2 |
| PSA_HAXA3 | UNITUS | 2012 | China | Shaanxi | Xi’an (Huxian) | *A. deliciosa* | Hayward | 5 | 10 | 3 | 7 | 3 | 2 | 6 | 7 | 12 | 5 | 8 | 8 | 2 |
| PSA_HAXA4 | UNITUS | 2012 | China | Shaanxi | Xi’an (Huxian) | *A. deliciosa* | Hayward | 6 | 12 | 3 | 4 | 4 | 2 | 4 | 6 | 16 | 5 | 5 | 15 | 2 |
| PSA_HAXA5 | UNITUS | 2012 | China | Shaanxi | Xi’an (Huxian) | *A. deliciosa* | Hayward | 5 | 10 | 3 | 7 | 3 | 2 | 6 | 7 | 12 | 5 | 8 | 8 | 2 |
| PSA_HAXA6 | UNITUS | 2012 | China | Shaanxi | Xi’an (Huxian) | *A. deliciosa* | Hayward | 5 | 10 | 3 | 7 | 3 | 2 | 6 | 7 | 12 | 5 | 8 | 8 | 2 |
| PSA_HAXA7 | UNITUS | 2012 | China | Shaanxi | Xi’an (Huxian) | *A. deliciosa* | Hayward | 5 | 10 | 3 | 7 | 3 | 2 | 6 | 7 | 12 | 5 | 8 | 8 | 2 |
| PSA_HAXA8 | UNITUS | 2012 | China | Shaanxi | Xi’an (Huxian) | *A. deliciosa* | Hayward | 5 | 10 | 3 | 7 | 3 | 2 | 6 | 7 | 12 | 5 | 8 | 8 | 2 |
| PSA_HYM1 | UNITUS | 2012 | China | Sichuan | Deyang (Mianzhu) | *A. chinensis* | Hong Yang | 6 | 12 | 3 | 4 | 4 | 2 | 4 | 6 | 16 | 5 | 5 | 15 | 2 |
| PSA_HYM2 | UNITUS | 2012 | China | Sichuan | Deyang (Mianzhu) | *A. chinensis* | Hong Yang | 5 | 10 | 3 | 7 | 4 | 2 | 6 | 7 | 12 | 5 | 8 | 8 | 2 |
| PSA_HYM3 | UNITUS | 2012 | China | Sichuan | Deyang (Mianzhu) | *A. chinensis* | Hong Yang | 5 | 10 | 3 | 7 | 4 | 2 | 6 | 7 | 12 | 5 | 8 | 8 | 2 |
| PSA_HYM4 | UNITUS | 2012 | China | Sichuan | Deyang (Mianzhu) | *A. chinensis* | Hong Yang | 5 | 10 | 3 | 7 | 4 | 2 | 6 | 7 | 12 | 5 | 8 | 8 | 2 |
| PSA_19439* | ICMP | 2011 | Chile | VII Reg. of Maule | - | *A. deliciosa* | new variety | 9 | 9 | 3 | 6 | 4 | 2 | 3 | 6 | 11 | 5 | 6 | 17 | 2 |
| PSA_19457 | ICMP | 2010 | Chile | VII Reg. of Maule | - | *A. deliciosa* | unknown | 9 | 9 | 3 | 6 | 4 | 2 | 3 | 6 | 11 | 5 | 6 | 17 | 2 |
| PSA_19438 | ICMP | 2011 | Chile | VII Reg. of Maule | - | *A. deliciosa* | new variety | 9 | 9 | 3 | 6 | 4 | 2 | 3 | 6 | 11 | 5 | 6 | 17 | 2 |
| PSA_19456 | ICMP | 2010 | Chile | VII Reg. of Maule | - | *A. chinensis* | unknown | 9 | 10 | 3 | 6 | 4 | 2 | 3 | 6 | 11 | 5 | 6 | 16 | 2 |
| PSA_Chile_1 | SAG-Chile | 2013 | Chile | VII Reg. of Maule | San Javier | *A. deliciosa* | Hayward | 9 | 10 | 3 | 6 | 4 | 2 | 3 | 6 | 11 | 5 | 6 | 16 | 2 |
| PSA_Chile_2 | SAG-Chile | 2013 | Chile | VIII Reg. of Bìo Bio | San Carlos | *A. deliciosa* | Hayward | 9 | 10 | 3 | 6 | 4 | 2 | 3 | 6 | 11 | 5 | 6 | 16 | 2 |
| PSA_Chile_3 | SAG-Chile | 2013 | Chile | VII Reg. of Maule | Longavi | *A. deliciosa* | Hayward | 9 | 10 | 3 | 6 | 4 | 2 | 3 | 6 | 11 | 5 | 6 | 16 | 2 |
| PSA_Chile_4 | SAG-Chile | 2013 | Chile | VII Reg. of Maule | Retiro | *A. deliciosa* | Hayward | 9 | 10 | 3 | 6 | 4 | 2 | 3 | 6 | 11 | 5 | 6 | 17 | 2 |
| PSA_Chile_5 | SAG-Chile | 2013 | Chile | VIII Reg. of Bìo Bio | San Carlos | *A. deliciosa* | Hayward | 9 | 8 | 3 | 6 | 4 | 2 | 3 | 6 | 11 | 5 | 6 | 16 | 2 |
| PSA_Chile_6 | SAG-Chile | 2013 | Chile | VII Reg. of Maule | Talca | *A. deliciosa* | Hayward | 9 | 10 | 3 | 6 | 4 | 2 | 3 | 6 | 11 | 5 | 6 | 16 | 2 |
| PSA_Chile_7 | SAG-Chile | 2013 | Chile | VII Reg. of Maule | Longavi | *A. chinensis* | KIS Y37 | 9 | 10 | 3 | 6 | 4 | 2 | 3 | 6 | 11 | 5 | 6 | 14 | 2 |
| PSA_Chile_8 | SAG-Chile | 2013 | Chile | VII Reg. of Maule | Retiro | *A. deliciosa* | Hayward | 9 | 10 | 3 | 6 | 4 | 2 | 3 | 6 | 11 | 5 | 6 | 16 | 2 |
| PSA_Chile_9 | SAG-Chile | 2013 | Chile | VIII Reg. of Bìo Bio | San Ignacio | *A. deliciosa* | Hayward | 9 | 10 | 3 | 6 | 4 | 2 | 3 | 6 | 11 | 5 | 6 | 16 | 2 |
| PSA_Chile_10 | SAG-Chile | 2013 | Chile | VII Reg. of Maule | Villa Alegre | *A. deliciosa* | Hayward | 9 | 10 | 3 | 7 | 4 | 2 | 3 | 6 | 11 | 5 | 6 | 8 | 2 |
| PSA_Chile_11 | SAG-Chile | 2013 | Chile | VII Reg. of Maule | San Javier | *A. deliciosa* | Hayward | 9 | 10 | 3 | 6 | 4 | 2 | 3 | 6 | 11 | 5 | 6 | 16 | 2 |
| PSA_Chile_12 | SAG-Chile | 2013 | Chile | VIII Reg. of Bìo Bio | Bulnes | *A. deliciosa* | Hayward | 9 | 10 | 3 | 6 | 4 | 2 | 3 | 6 | 11 | 5 | 6 | 16 | 2 |
| PSA_Chile_13 | SAG-Chile | 2014 | Chile | VII Reg. of Maule | Molina | *A. deliciosa* | Matua | 9 | 10 | 3 | 6 | 4 | 2 | 3 | 6 | 11 | 5 | 6 | 16 | 2 |
| PSA_Chile_14 | SAG-Chile | 2013 | Chile | VIII Reg. of Bìo Bio | San Ignacio | *A. deliciosa* | Hayward | 9 | 10 | 3 | 6 | 4 | 2 | 3 | 6 | 10 | 5 | 6 | 16 | 2 |
| PSA_Chile_15 | SAG-Chile | 2013 | Chile | VII Reg. of Maule | Retiro | *A. deliciosa* | Hayward | 9 | 10 | 3 | 6 | 4 | 2 | 3 | 6 | 11 | 5 | 6 | 17 | 2 |
| PSA_Chile_16 | SAG-Chile | 2013 | Chile | VII Reg. of Maule | Colbun | *A. deliciosa* | Hayward | 9 | 10 | 3 | 6 | 4 | 2 | 3 | 6 | 11 | 5 | 6 | 16 | 2 |
| PSA_Chile_17 | SAG-Chile | 2013 | Chile | VIII Reg. of Bìo Bio | San Ignacio | *A. deliciosa* | Hayward | 9 | 10 | 3 | 6 | 4 | 2 | 3 | 6 | 11 | 5 | 6 | 16 | 2 |
| PSA_Chile_18 | SAG-Chile | 2013 | Chile | VIII Reg. of Bìo Bio | Bulnes | *A. deliciosa* | Hayward | 9 | 10 | 3 | 6 | 4 | 2 | 3 | 6 | 11 | 5 | 6 | 16 | 2 |
| PSA_Chile_19 | SAG-Chile | 2013 | Chile | VII Reg. of Maule | Villa Alegre | *A. deliciosa* | Hayward | 9 | 10 | 3 | 6 | 4 | 2 | 3 | 6 | 9 | 5 | 6 | 17 | 2 |
| PSA_Chile_20 | SAG-Chile | 2013 | Chile | VII Reg. of Maule | Retiro | *A. deliciosa* | Hayward | 9 | 10 | 3 | 6 | 4 | 2 | 3 | 6 | 11 | 5 | 6 | 18 | 2 |
| PSA_Chile_21 | SAG-Chile | 2013 | Chile | VIII Reg. of Bìo Bio | Bulnes | *A. deliciosa* | Hayward | 9 | 10 | 3 | 6 | 4 | 2 | 3 | 6 | 11 | 5 | 6 | 16 | 2 |
| PSA_Chile_22 | SAG-Chile | 2013 | Chile | VIII Reg. of Bìo Bio | San Ignacio | *A. deliciosa* | Hayward | 9 | 9 | 3 | 6 | 4 | 2 | 3 | 6 | 11 | 5 | 6 | 16 | 2 |
| PSA_Chile_23 | SAG-Chile | 2014 | Chile | VII Reg. of Maule | Molina | *A. deliciosa* | Matua | 9 | 10 | 3 | 6 | 4 | 2 | 3 | 6 | 9 | 5 | 6 | 17 | 2 |
| PSA_Chile_24 | SAG-Chile | 2013 | Chile | VII Reg. of Maule | Yerbas Buenas | *A. deliciosa* | Hayward | 9 | 10 | 3 | 6 | 4 | 2 | 3 | 6 | 11 | 5 | 6 | 16 | 2 |
| PSA_Chile_25 | SAG-Chile | 2013 | Chile | VIII Reg. of Bìo Bio | San Carlos | *A. deliciosa* | Hayward | 9 | 10 | 3 | 6 | 4 | 2 | 3 | 6 | 11 | 5 | 6 | 16 | 2 |

^†^strains used as preliminary “diversity panel” to assess VNTR loci variability

*strains whose WGS sequences are available on NCBI database
